# A systematic review on the impact of gastrointestinal microbiota composition and function on cognition in healthy infants and children

**DOI:** 10.3389/fnins.2023.1171970

**Published:** 2023-06-14

**Authors:** Arden L. McMath, Miriam Aguilar-Lopez, Corinne N. Cannavale, Naiman A. Khan, Sharon M. Donovan

**Affiliations:** ^1^Division of Nutritional Sciences, University of Illinois Urbana-Champaign, Urbana, IL, United States; ^2^Department of Pathology and Immunology, Baylor College of Medicine, Houston, TX, United States; ^3^Texas Children’s Microbiome Center, Department of Pathology, Texas Children’s Hospital, Houston, TX, United States; ^4^Department of Kinesiology and Community Health, University of Illinois Urbana-Champaign, Urbana, IL, United States; ^5^Neuroscience Program, University of Illinois Urbana-Champaign, Champaign, IL, United States; ^6^Beckman Institute for Advanced Science and Technology, University of Illinois Urbana-Champaign, Champaign, IL, United States; ^7^Department of Food Science and Human Nutrition, University of Illinois Urbana-Champaign, Urbana, IL, United States

**Keywords:** gut microbiome, infancy, childhood, behavior, cognition

## Abstract

Evidence from animal models or children with neurodevelopmental disorders has implicated the gut microbiome (GM) in neurocognitive development. However, even subclinical impairement of cognition can have negative consequences, as cognition serves as the foundation for skills necessary to succeed in school, vocation and socially. The present study aims to identify gut microbiome characteristics or changes in gut microbiome characteristics that consistently associate with cognitive outcomes in healthy, neurotypical infants and children. Of the 1,520 articles identified in the search, 23 were included in qualitative synthesis after applying exclusion criteria. Most studies were cross-sectional and focused on behavior or motor and language skills. *Bifidobacterium, Bacteroides*, Clostridia, *Prevotella*, and *Roseburia* were related to these aspects of cognition across several studies. While these results support the role of GM in cognitive development, higher quality studies focused on more complex cognition are needed to understand the extent to which the GM contributes to cognitive development.

## 1. Introduction

Studies exploring the contribution of the gut microbiome, a complex ecosystem of microbes and their corresponding genomes residing in the gastrointestinal tract (Turnbaugh et al., 2007), to human cognitive development have emerged in recent years. These studies were initially driven by observations of altered microbial colonization in psychological disorders (Valles-Colomer et al., 2019), altered brain structure (Erny et al., 2015) and behavior (Clarke et al., 2013) in germ-free mice models, as well as the parallel developmental trajectories of the intestinal microbiota and nervous system (Borre et al., 2014). Longitudinal magnetic resonance imaging (MRI) studies suggest that brain regions responsible for development of cognitive functions, such as the prefrontal cortex, undergo dynamic changes from birth up through adolescence (Casey et al., 2005). The intestinal microbiome appears to be integral to these processes directly, through production of metabolites that cross the blood-brain barrier (Blaak et al., 2020) and indirectly, through regulation of the immune system or maintenance of gut barrier function (Peng et al., 2009; Tommaso et al., 2021).

To date, systematic reviews have focused on gut microbiome comparisons between children with and without neurodevelopmental disabilities [i.e., attention-deficit/hyperactivity disorder (ADHD), autism spectrum disorder (ASD), learning disabilities] (Bundgaard-Nielsen et al., 2020; Iglesias-Vázquez et al., 2020). Poor cognition is a hallmark of these disabilities, and cognition can be defined as “the processes an organism uses to organize information. This includes acquiring information (sensation and perception), selecting (attention), communicating (language, numbers), representing (understanding), and retaining (memory) information, and using it to guide behavior (reasoning and coordination of motor outputs)”(Sela and Lavidor, 2014). While reviews focused on children with neurodevelopmental disabilities report differences in gut microbiome composition (Bundgaard-Nielsen et al., 2020; Iglesias-Vázquez et al., 2020), many studies included are cross-sectional or observational, making it difficult to determine whether other symptomology (i.e., picky eating, commonly observed in ASD) impact the gut microbiome, rather than the reverse. By studying the gut microbiome and cognition in children defined as neurotypical, it is possible to develop a more comprehensive understanding of the directionality as well as moderating factors involved in the interactions between the gut microbiome and cognition throughout childhood. Additionally, even in sub-clinical impairment, cognition in childhood is relevant to various aspects of life, including academic achievement (Blair and Razza, 2007) and later physical (Moffitt et al., 2011) and mental health (Tangney et al., 2004).

The gastrointestinal microbiota is a promising avenue for interventions aimed at optimizing cognitive development, given the robust variations observed in microbial composition and function by diet, even in children (Dinsmoor et al., 2021). Indeed, work has already begun in this area; in a recent review of the effects of pre and probiotic supplementation and fecal microbiota transplantation in adults, five studies out of eight observed improvements in cognitive function (Baldi et al., 2021). In children, much of the human research on gut-microbiome-brain interactions are cross-sectional in nature and focused on infant temperament (Aatsinki et al., 2019) and early childhood behavior (Flannery et al., 2020), both outcomes of cognition (Sela and Lavidor, 2014). Few studies investigate the development of complex cognition directly during childhood (Sordillo et al., 2019; Streit et al., 2021).

Therefore, this systematic review aims to identify gut microbiome characteristics or changes in gut microbiome characteristics (through intervention) that consistently associate with cognitive outcomes (cross-sectionally or longitudinally) in healthy, neurotypical infants and children. Given the scope of the term “cognition,” a wide variety of cognitive outcomes were included (see Table 1 for definitions of cognitive outcomes included in the present review). Understanding gut microbiome relationships with cognitive abilities in neurotypically developing children will allow us to better understand how we might promote optimal cognitive development and lessen the burden of neurodevelopmental disorders.

**TABLE 1:** Definitions of cognition, temperament, and subscales of related cognitive outcomes commonly observed in studies included in the present review.

Cognition	“The processes an organism uses to organize information. This includes acquiring information (sensation and perception), selecting (attention), communicating (language, numbers), representing (understanding) and retaining (memory) information, and using it to guide behavior (reasoning and coordination of motor outputs)” (Sela and Lavidor, 2014).
Temperament	“Individual differences in emotional, motor, and attentional reactivity measured by latency, intensity, and recovery of response, and self-regulation processes…that modulate reactivity” (Rothbart, 2007).
Regulation/orienting	Aspect of temperament that includes subscales of perceptual sensitivity, low-intensity pleasure, cuddliness, duration of orienting, soothability; (Rothbart, 2007) overlapping with adaptive behaviors and effortful control (Nigg, 2017).
Effortful control (i.e., top-down regulation)	Aspect of temperament that includes subscales of inhibitory control, attentional control, low-intensity pleasure, perceptual sensitivity; (Rothbart, 2007) low effortful control is related to increased risk for poor mental health characteristics and neurodevelopmental outcomes; (Kostyrka-Allchorne et al., 2020) overlapping scales with regulation/orienting and has a bidirectional relationship with reactive control (Nigg, 2017).
Extraversion/surgency (i.e., positive affect)	Aspect of temperament that includes subscales of activity level, smiling and laughter, high-intensity pleasure, approach, vocal reactivity, impulsivity, sociability, positive anticipation; (Rothbart, 2007) predictive of better self-regulation, lower depressive and ASD symptoms (Komsi et al., 2006; Kostyrka-Allchorne et al., 2020).
Negative affect	Aspect of temperament that includes discomfort, fear, sadness, soothability, and frustration; (Rothbart, 2007) those with higher negative affect are at greater risk for poor mental health characteristics and neurodevelopmental outcomes (Kostyrka-Allchorne et al., 2020).
Behavioral problems	Includes subscales of emotional reactivity, anger-frustration, and inhibitory control; (Achenbach, 1999) results partially from lack of bottom-up regulation, reactive control or behavioral inhibition (Nigg, 2017).
Internalizing behavioral problems	Includes subscales of anxiety, depression, and somatic complaints (Achenbach, 1999).
Externalizing behavioral problems	Includes subscales of impulsivity, rule-breaking, and aggression (Achenbach, 1999).
Motor skills	Uses measures of visual tracking, reaching, object manipulation, grasping, functional hand skills, sensory integration, static positioning, locomotion, quality of movement, balance, motor planning and perceptual-motor integrating to measure fine (associated with prehension, perceptual-motor integration, motor planning, motor speed) and gross motor skills; (Bayley, 2006; Squires et al., 2009) overlapping with performance skills, which includes visual-spatial orienting and fine motor manipulation (Bayley, 2006; Squires et al., 2009).
Language skills	Measures expressive and receptive communication via preverbal behaviors and vocabulary development; expressive communication describes babbling, gesturing, joint referencing, turn taking, naming of objects, pictures and actions, and morpho-syntactic development (i.e., use of plurals or verb tense); receptive communication describes ability to identify referenced objects or pictures, use of pronouns and predispositions, understanding of morphological markers (i.e., plurals and tenses); (Bayley, 2006; Squires et al., 2009) related to personal-social skills (Squires et al., 2009).

## 2. Methods

The present review was registered in the PROSPERO database (CRD42021268887) and performed according to the guidelines for the Preferred Reporting Items for Systematic Reviews and Meta-analysis Protocols (PRISMA) (Moher et al., 2016).

### 2.1. Data sources and search strategy

PubMed, Scopus, Web of Science and Cochrane Library were searched for studies related to the impact of gut microbiome composition and function on cognition in infants, children and adolescents in August of 2021. Studies were restricted to English language only. Search terms included: “infant,” “child,” “children,” “childhood,” “toddler,” “early life,” “gastrointestinal microbiome,” “intestinal microbiome,” “gastrointestinal microbiota,” “intestinal microbiota,” “gastrointestinal microbes,” “intestinal microbes,” “gut microbiome,” “gut microbiota,” “gut microbes,” “fecal microbiome,” “fecal microbiota,” “fecal microbes,” “metagenome,” “metabolome,” “metabolite,” “short-chain-fatty acid,” “volatile fatty acid,” “cognitive function,” “cognitive control,” “cognition,” “self-regulation,” “self-control,” “executive function,” “inhibition,” “inhibitory control,” “attention,” “fMRI,” “interference control,” “working memory,” “short-term memory,” “long-term memory,” “episodic memory,” “spatial memory,” “cognitive flexibility,” “task switching,” “emotion,” “temperament,” “negative affect,” “positive affect,” “mood,” “neural development,” “neural growth,” “neurogenesis,” “prefrontal cortex,” “dorsolateral prefrontal cortex,” “anterior cingulate cortex,” “cerebral cortex,” “hippocampus,” “amygdala,” “basal ganglia,” “striatum,” “brain.”

Studies with the following terms in the title were excluded: “mice,” “mouse,” “murine,” “rat,” “rodent,” “piglet,” “pig,” “swine,” “monkey,” “rhesus macaque,” “hamsters,” “chicken,” “quail,” “animal,” “preterm,” “IBS,” “IBD,” “ulcerative colitis,” “ADHD,” “autism,” “autistic,” “colic,” “diarrhea,” “deficiency,” “disorder,” “disease,” “disability,” “asthma,” “syndrome,” “epilepsy,” “gabapentin,” “allergy,” “cancer,” “leukeaemia,” “leukemia,” “oncology,” “tumor,” “diabetes,” “hyperglycemia,” “insulin resistance,” “undernutrition,” “malnutrition,” “arthritis,” “schizophrenia,” “drug abuse,” “drug misuse,” “alcohol abuse,” “alcohol misuse,” “substance abuse,” “defect,” “acidemia,” “infection,” “necrotizing enterocolitis,” “necrotising enterocolitis,” “injury,” “illness,” “anorexic,” “bulimic,” “eating disorder,” “review,” “protocol,” “letter,” “commentary,” and “editorial.” Studies with the following terms in the title or abstract were excluded: “surgery,” “migraine,” “seizure,” “anti-convulsant,” “anticonvulsant,” “antiepileptic,” “meta-analysis,” “systematic review,” and “review of the literature.” Studies with the following index terms were excluded: “rodentia,” “swine,” “tarsii,” “platyrrhini,” “hylobatidae,” and “cercopithecidae.”

### 2.2. Study selection

Eligible studies included those related to the effect or association of the gastrointestinal microbiota on cognition and its related constructs or measures in healthy human infants (birth to <2 years old) and children (2–12 years old). Randomized controlled trials, cross-sectional, longitudinal, or case-control studies were included. Narrative reviews, systematic reviews, meta-analyses, animal and *in vitro* studies were excluded. Inclusion of only healthy and neurotypically-developing humans was observed by excluding studies focused on individuals with diagnosed neurodevelopmental disorders (e.g., ASD, ADHD), acute or chronic diseases or born preterm. Two authors (ALM and MAL) separately searched the predetermined databases using the search strategy designed *a priori* for this review. These articles were retained or discarded according the inclusion and exclusion criteria. Any disagreements between initial reviewers were resolved by a third party (SMD).

### 2.3. Data collection process

Per the guidance of an adapted data collection form from the Cochrane Handbook for Systematic Reviews, (“The Cochrane Collaboration’s Tool for Assessing Risk of Bias in Randomized Trials,”) the following data was extracted from each of the studies included in the review: author, year of publication, journal, geographic location of the study, study design, method of participant recruitment, exclusion criteria, sample size, duration of follow-up or intervention, study aims and hypotheses, baseline imbalances, withdrawals and exclusions, age at each visit, sex and race/ethnicity distribution, study funding sources, and possible conflicts of interest. Additionally, due to the relevance for quality of the included studies, method of gut microbiota assessment, microbial DNA extraction method, 16S rRNA variable region, sequencing platform, alpha diversity, beta diversity, taxonomy, other microbiota related outcomes, and neurocognitive outcome measures and methods were extracted from each study included in the review.

### 2.4. Quality of evidence and risk of bias assessment

Assessment of study quality was conducted using the National Institute for Health (NIH) Quality Assessment Tool for observational, cross-sectional studies and controlled intervention studies (Higgins et al., 2011). Briefly, quality assessment using the NIH Quality Assessment Tool for cross-sectional and observational studies involves 14 criteria, which are assigned yes, no, not specified, or not applicable. Quality of studies are denoted by allocation poor (0–4 yes out of 14 questions), fair (5–10 yes out of 14 questions) or good (11–14 yes out of 14 questions). Note, for the criteria “were key potential confounding variables measured and adjusted statistically for their impact on the relationship?”, it was necessary to predefine confounding variables; thus, studies controlling for antibiotic usage and any aspect of child diet (e.g., breastfeeding, timing of introduction to solids, fruit and vegetable intake) via exclusion from the study or statistical adjustment were allocated a “yes.” Studies not controlling for antibiotic exposure or any aspect of diet received a “no.” The NIH Quality Assessment tool for controlled intervention studies also includes 14 criteria that are assigned a yes, no, not specified or not applicable. Studies were assigned an over allocation of poor, fair or good, based on perception of potential for significant risk of bias. For example, a study demonstrating high risk of bias (i.e., employed a per-protocol analysis or lacked blinding of treatment to participants or researchers) would be allocated a poor overall quality rating.

## 3. Results

### 3.1. Study selection

**FIGURE 1:**
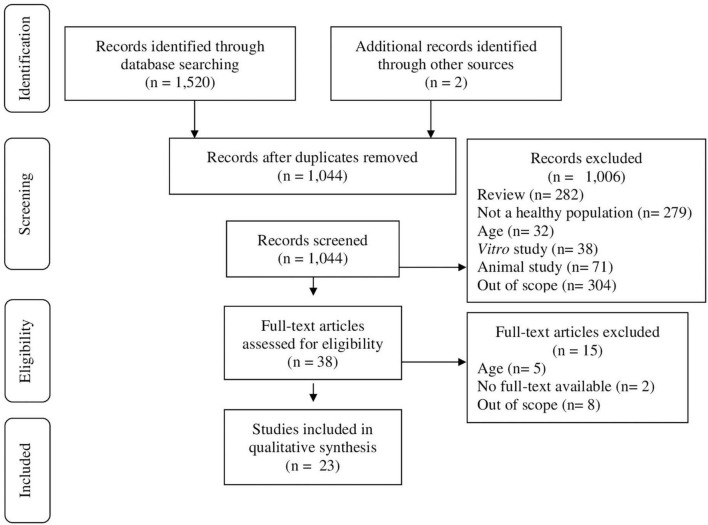
Preferred Reporting Items for Systematic Reviews and Meta-analysis Protocol (PRISMA) flow diagram of search strategy.

A total of 1,520 articles were identified. After removal of duplicates and after screen by title and abstract for inclusion/exclusion criteria, 1,044 studies remained. Two studies were identified through alternative sources. 1,006 articles were excluded based on it not being a primary research article (*n* = 282), not including a healthy population (*n* = 279), outside the age range (*n* = 32), *in vitro* (*n* = 38) or animal (*n* = 71) study, or out of scope (*n* = 304). Full text of 38 articles were screened, upon which 15 articles were excluded, based on inclusion of adults (and no separated analysis for adults and children) (*n* = 5), no full-text available (*n* = 2), or out of scope for aims of the present review (*n* = 8). A total of 23 studies were included in the present review (Figure 1).

### 3.2. Study characteristics

Characteristics of the included studies are shown in Supplementary Table 1. The majority of studies (20/22) were observational. Roughly half of these were cross-sectional (9/20), while the rest were longitudinal studies exploring associations between microbiota earlier in life (first pass meconium samples to 12 months) with later neurocognitive outcomes (6 months to 5 years). Two studies were randomized-controlled trials (RCT); interventions included feeding an sn-2 palmitate-rich infant formula for the first 16 weeks of life (Wu et al., 2021) and a 12 weeks outdoor play program in preschoolers (Sobko et al., 2020). Several studies used the same cognitive assessment tools, including the Ages and Stages Questionnaire (Squires et al., 2009) (*n* = 2), Bayley Scale of Infant Development (2nd and 3rd editions) (Bayley, 1993, 2006) (*n* = 3), Infant Behavior Questionnaire (Putnam et al., 2014) (*n* = 5), Mullen scales of early learning (Mullen, 1995) (*n* = 2), and the Child Behavior Checklist (Achenbach, 1999) (*n* = 3). Results pertaining to child temperament and behavior are found in Supplementary Table 2, whereas results pertaining to basic and higher order cognitive functions are in Supplementary Table 3.

Several groups of studies included data from the same longitudinal cohorts followed in the US (Carlson et al., 2018, 2021; Gao et al., 2018) and Finland (Aatsinki et al., 2019, 2020), and therefore had overlapping participants. Studies including older children were with age ranges of 5–7 years old (Flannery et al., 2020), 5–11 years old (Callaghan et al., 2019), and 8–11 years old (Michels et al., 2019). The remaining studies (*n* = 19) included ages 5 years old and below, with the majority being <2 years old. As a result, study results were reported and discussed by infants (cognitive assessments conducted at 1 year old and younger), pre-school children (>1 to <5 years old) and school-aged children (5 to <12 years old). Three studies explored associations between gut metagenomes and cognitive outcomes (Sobko et al., 2016; Flannery et al., 2020; Kelsey et al., 2021). One study explored relationships of cognitive outcomes with the microbiome metabolites, volatile fatty acids (VFAs) (Loughman et al., 2020). Two studies measured functional connectivity of the amygdala (Gao et al., 2019), prefrontal cortex, and parietal cortex (Kelsey et al., 2021), and two studies measured volumetric differences of the prefrontal cortex, amygdala, hippocampus, total gray and white matter, cerebrospinal fluid, intracranium and lateral ventricle utilizing structural MRI (Carlson et al., 2018, 2021; Supplementary Table 1).

All studies measured gut microbiota using next-generation sequencing technologies, mostly targeting the V1-V2 (*n* = 5) (Carlson et al., 2018, 2021; Callaghan et al., 2019; Gao et al., 2019; Acuna et al., 2021), V3-V4 (*n* = 7) (Michels et al., 2019; Sobko et al., 2020; Wang et al., 2020; Fox et al., 2021; Rothenberg et al., 2021; Streit et al., 2021; Guzzardi et al., 2022), or V4 (*n* = 5) (Aatsinki et al., 2019, 2020; Loughman et al., 2020; Tamana et al., 2021; Wu et al., 2021) regions of the 16S rRNA bacterial gene. Several studies also used *Bifidobacterium* qPCR primers with next-generation sequencing (Carlson et al., 2018, 2021; Gao et al., 2018). Two studies used whole genome shotgun sequencing (Flannery et al., 2020; Kelsey et al., 2021). All studies used Illumina technology sequencing platforms, except for two that used Roche 454 GC FLX Titanium (Christian et al., 2015; Sordillo et al., 2019).

### 3.3. Quality of the evidence

Only one study received a “poor” summary of quality rating, while most studies received a “fair” (14/20) or “good” (5/20) rating using the NIH Quality Assessment tool for observational and cross-sectional studies. The primary sources of bias included lack of sample size justification or power description, and the inability to confer directionality or relationships between gut microbiome and cognition over time (due to cross-sectional study designs). Most observational studies conducted in children 7 years old and below explored sex, socioeconomic variables, breastfeeding status or duration and mode of delivery posteriori for inclusion as covariates, except for two studies which did not consider all of them (Zhang et al., 2021; Guzzardi et al., 2022). However, several studies did not adjust for key covariates, such as antibiotic usage (Supplementary Table 4; Christian et al., 2015; Callaghan et al., 2019; Wang et al., 2020; Acuna et al., 2021; Rothenberg et al., 2021; Guzzardi et al., 2022). For the two studies assessed via the NIH Quality Assessment Tool for controlled intervention studies, one was rated good (Sobko et al., 2020) and the other of poor (Wu et al., 2021) summary quality (Supplementary Table 5). Wu et al. (2021) did not specify whether participants and researchers were blind to their infant formula treatment versus control. Further, this study was unable to fully randomize their study participants between the three groups (control infant formula vs. sn-2 palmitate-rich infant formula vs. exclusively breastfed) for ethical reasons (Wu et al., 2021). Potentially due to inability for appropriate randomization, the formula-fed groups had significantly lower maternal education status and vaginal deliveries compared to each other and to the breastfed group. Of note, these characteristics have been previously associated with gut microbiome characteristics (Borre et al., 2014) and neurodevelopmental outcomes (Casey et al., 2005).

### 3.4. Infants (cognitive assessment at 1 year old and younger)

#### 3.4.1. Alpha diversity

Studies exploring cognition in infants was largely focused on infant temperament and was not associated with alpha diversity, with the exception of some for measures of alpha diversity that include evenness of microbial species. Orienting/regulation was measured in four studies in infants; of these, three assessed relationships with alpha diversity and found no associations between Chao1 (a measure of species richness) or Shannon Diversity (a measure of richness and evenness) (Aatsinki et al., 2019; Fox et al., 2021; Kelsey et al., 2021). Negative affectivity was measured in four studies of infants, while two found no associations between parent-reported negative affectivity and Shannon Diversity or Chao1 (Wang et al., 2020; Kelsey et al., 2021). The other two studies found negative associations between alpha diversity and negative affect. Specifically, principle component-derived clusters of microbial alpha diversity (with the cluster mostly describing taxonomic evenness) at 1 month old was negatively associated with laboratory-assessed non-social fear behavior at 1 year. This study also found 1 month old and 1 year old alpha diversity were not associated with 1 year parent-reported fear (Carlson et al., 2021). Microbial Shannon Diversity at 2.5 months old, but not Chao1 index, was also negatively associated with negative affect and fear reactivity at 6 months old (Aatsinki et al., 2019). Lastly, five studies conducted in infants measured surgency, of which four assessed relationships with Shannon Diversity and Chao1 and found no relationships (Aatsinki et al., 2019; Wang et al., 2020; Fox et al., 2021; Kelsey et al., 2021).

#### 3.4.2. Beta diversity

Most studies conducted in infants utilized non-phylogenetic distance metrics to measure beta diversity, with the exception of one (Carlson et al., 2021). Results for relationships between beta diversity and temperament was mixed. One study found no longitudinal (1–3 weeks, 2, 6 months old) or cross-sectional (12 months old) relationships with 12 month old regulation or negative affect, except for an association between beta diversity and the sadness subscale of negative affect (Fox et al., 2021). This study was also one of two to assess relationships between surgency/positive emotions and beta diversity in infants; they found significant associations between 1 and 3 weeks beta diversity and 12 month old surgency and its subscales, approach, high-intensity pleasure and smiling/laughter, even after adjustment for breastfeeding duration and child sex. However, 2-, 6-, and 12 month old beta diversity was not associated with 12 month old surgency or its subscales (Fox et al., 2021).

Another study in infants demonstrated that, upon comparison to a *Bifidobacterium/Enterobacteriaeae*-dominant cluster at 2.5 months old, those with a *Bacteroide*s- or *Veillonella dispar*-dominant community structure had poorer regulation and lower surgency at 6 months old (Aatsinki et al., 2019). The latter association with surgency failed to remain significant after adjusting for sex and mode of delivery, however. Lastly, this study found 2.5 months old beta diversity was not associated with 6 months old negative affect (Aatsinki et al., 2019).

Weighted UniFrac (but not Unweighted) was associated with non-social fear behavior at 1 year old-of-age, measured via a laboratory-based assessment that involved recording of child fear response to a research assistant wearing four different masks (horse, apple, monkey, alien). Researchers coded the task fear response by observing facial, vocal and bodily fear reactions. Specifically, those with a community structure characterized by higher abundances of *Veillonella, Dialister*, unnamed genus of *Clostridiales, Bifidobacterium*, and *Lactobacillus*, and by lower abundance of *Bacteroides* had higher non-social fear behavior. Of note, in this study there was no association between beta diversity at 1 month old with any measure of negative affect, nor was there an association between 1 year old beta diversity, laboratory-assessed social fear behavior or parent-reported negative affectivity (Carlson et al., 2021).

#### 3.4.3. Taxonomy

Uncharacterized *Bifidobacterium* OTUs (Aatsinki et al., 2019; Wang et al., 2020) and other species of *Bifidobacterium*, such as *B. pseudocatenulatum* and *B. catenulatum* (using WGS) (Kelsey et al., 2021) were positively associated with regulation/orienting aspect of infant temperament, which is also related to effortful control (Rothbart et al., 2004). Other studies showed *Bifidobacterium* was positively associated with extraverted infant temperament (Fox et al., 2021), although one study found this relationship was specific to males only (Aatsinki et al., 2019). One study evaluated associations of Bifidobacteria abundances with motor, problem-solving, comunication, personal and social skills, finding no relationships at 16 weeks-of-age. However, the study did find that higher fecal Bifidobacteria abundance was associated with lower odds of at least one of the aforementioned domains scoring close to the typical development threshold (Wu et al., 2021).

Aatsinki et al. (2019) demonstrated that female infants with higher *Veillonella parvula* and *Veillonella dispar* had lower fear reactivity at 6 months old (subscale of negative affect). This study also found that, in male infants, these same *Veillonella* spp. were negatively associated with regulation/orienting and, among the total sample, *Veillonella dispar* was related to lower surgency (Aatsinki et al., 2019). *Lactobacillus* was also relevant for this age-group, as it was associated with lower behavioral problems at age 3 years old (Zhang et al., 2021), negative affect at age 1 year old (Fox et al., 2021), and higher surgency at 6 months old (Aatsinki et al., 2019).

#### 3.4.4. Metabolomics and metabolites

One study evaluated relationships of infant temperament with virulence factors, resistance genes or genes contributing to biology of an organism at molecular, cellular or organism levels, and found no significant associations (Kelsey et al., 2021).

### 3.5. Preschool-ages (cognitive assessments at ≥1 to 4 years old)

#### 3.5.1. Alpha diversity

Several aspects of temperament had sex-dependent relationships with alpha diversity in 24 months old children; Shannon Diversity was negatively associated with effortful control in females, but not males. Additionally, surgency and its subscale, high-intensity pleasure, were associated with phylogenetic diversity in females only. However, negative affect aspect of temperament was not associated with Shannon Diversity or Chao1 in this study for either sex (Christian et al., 2015).

Conversely, other aspects of cognition that overlap with negative affect, behavioral problems and perceived stress, were related to microbial alpha diversity. Although gut microbiome measured at younger timepoints (2 and 6 months old) was not related, a trending (*p* = 0.087) association between higher 12 months old Shannon Diversity and elevated behavioral problems at 2 years old was observed in one study (Loughman et al., 2020). An RCT employing a 12 weeks outdoor play intervention found that, while alpha diversity did not change throughout the intervention, lower perceived stress was associated with higher Chao1, Shannon Diversity, Simpson Diversity and diversity of Bacteroidetes (Sobko et al., 2020). Additionally, preschool-aged children in the outdoor play intervention group who experienced significant decreases in their perceived stress post-intervention had significantly higher Chao1 compared to non-responders of the intervention group (Sobko et al., 2020).

Primary functions measured most often throughout these studies included language and motor skills. Of the nine studies measuring these basic skills, four explored relationships with alpha diversity. Faith’s Phylogenetic Diversity was negatively associated with 4 year old (Streit et al., 2021) and 2 year old (Carlson et al., 2018) language skills. At 1 year, Chao1 and Observed Species measures were also negatively associated with 2 year old language skills. Carlson et al. (2018) also demonstrated that lower Chao1, Observed Species and Phylogenetic Diversity at 1 year old were related to greater change scores for expressive and receptive language skills between 1- and 2 years old, even after adjustment for beta diversity clusters as a covariate. Three studies found no relationship between Shannon Diversity and language/communication skills (Sordillo et al., 2019; Acuna et al., 2021; Streit et al., 2021).

Four of these studies assessed relationships between motor skills and alpha diversity measures, finding that Shannon Diversity (Carlson et al., 2018; Sordillo et al., 2019; Acuna et al., 2021; Rothenberg et al., 2021) and Faith’s Phylogenetic Diversity (Carlson et al., 2018; Acuna et al., 2021; Rothenberg et al., 2021) were not associated with motor skills. Other measures of alpha diversity, such as Pielou’s measure of evenness (Rothenberg et al., 2021) and Observed Species (Carlson et al., 2018), were also not related to motor skills. One study found that higher Chao1, Observed Species and Shannon Index, but not Phylogenetic Diversity, were associated with lower visual reception (Carlson et al., 2018). No other studies measured visual abilities directly.

Higher order functions were highly heterogenous, including adaptive behavior, personal and social skills, practical reasoning skills, problem-solving abilities and general cognitive skills. Additionally, five studies explored relationships with gut microbiome and a cognitive skills composite, compromising of language, motor and other cognitive abilities (Carlson et al., 2018; Acuna et al., 2021; Rothenberg et al., 2021; Streit et al., 2021; Guzzardi et al., 2022). Four studies with a cognitive composite assessed relationships with alpha diversity, with two finding no associations with Shannon Diversity or Faith’s Phylogenetic Diversity (Acuna et al., 2021; Rothenberg et al., 2021). At 2 years old, early learning composite was negatively associated with multiple measures of 1 year old alpha diversity. However, this was abrogated upon inclusion of beta diversity as a covariate in the model. Streit et al. (2021) found a negative association before, but not after adjustment for multiple comparisons, between Faith’s Phylogenetic Diversity and 4 year full-scale intelligence quotient (IQ) (Streit et al., 2021). Other higher order functions, problem-solving, personal, social, and performance skills, were not associated with multiple measures of alpha diversity (Sordillo et al., 2019; Streit et al., 2021).

#### 3.5.2. Beta diversity

Beta diversity assessed via PCoA of Bray Curtis distances or Weighted and Unweighted UniFrac distances were not related to parent-reported effortful control in 2- or ∼6 year olds, respectively (Christian et al., 2015; Flannery et al., 2020).

As was the case with alpha diversity relationships to cognition, Christian et al. (2015) found several aspects of temperament had sex-dependent relationships with beta diversity at 24 months old; the fear subscale of negative affectivity in females only, while surgency in males only were associated with Unweighted, but not Weighted, UniFrac distances at 24 months old. Related to negative affect, anger frequency was also associated with beta diversity in preschool-aged children (Sobko et al., 2020).

Five studies assessed relationships between beta diversity and language skills, and three demonstrated a significant relationship. Receptive and expressive language at age two and change in scores from 1 to 2 years old were highest in those with a community structure characterized by high abundance of *Bacteroides* at 1 year old, whereas those with a *Ruminococcaceae*-dominant, followed by *Faecalibacterium* (of the Clostridia class)-dominant microbiome had the lowest scores (Carlson et al., 2018). Similarly, those with 12 months old Bacteroidetes-dominant community structure had the highest 2 year old language scores and change scores from 1 to 2 years old, followed by *Firmicutes*-dominant and then Proteobacteria-dominant clusters (Tamana et al., 2021). Neither of these studies found a relationship between beta diversity and language skills at 12 months old (Carlson et al., 2018; Tamana et al., 2021). Sordillo et al. (2019) also showed that community structure defined by higher abundance of *Lachnospiraceae* and unclassified *Clostridiales* (both of the Clostridia class), and low abundances of *Bacteroides*, was associated with lower communication skills (Sordillo et al., 2019).

Seven studies explored relationships between beta diversity and motor skills. Two studies found relationships with fine, rather than gross, motor skills. Another study in 18 months old children demonstrated an association between both PCA clustering (weighted UniFrac) and genus-level enterotypes with fine motor skills. Specifically, those with a community structure characterized by higher abundances of Firmicutes such as *Lachnospiracea_incertae_sedis, unclass_Lachnospiraceae, Streptococcus*, and *Blautia*, as well as *Fusicatenibacter* and *Anaerostipes*, had higher fine motor skills compared to those with an enterotype characterized by high abundances of *Bacteroides* such as *Clostridium* XIVa and *Parabacteroides* (Acuna et al., 2021). In a longitudinal study, Sordillo et al. (2019) also demonstrated a specific relationship with fine, but not gross, motor skills and the gut microbiota, observing that a community structure characterized by higher abundance of *Bacteroides* and lower abundances of *Escherichia/Shigella* and *Bifidobacterium* at 3–6 months old was associated with lower fine motor skills at 3 years old (Sordillo et al., 2019). Other studies employing a longitudinal approach, comparing early gut microbiota to both present and later motor skills, found no relationships throughout early childhood (Carlson et al., 2018; Guzzardi et al., 2022). Similarly, Tamana and collaborators found no relationship between 4 months old beta diversity and 1 or 2 year old motor skills, nor was 1 year old beta diversity associated with 1 year old motor skills. However, this study demonstrated a longitudinal relationship between 1 year old Bacteroidetes-dominant community structure and better motor skills at age two, in comparison to a Firmicutes or Proteobacteria-dominant (lowest scoring for motor skills) community structure (Tamana et al., 2021). Lastly, at 3 years old, motor skills were better in those with a community structure characterized by high abundances of *Faecalibacterium, Clostridium* cluster XIVa, *Gemmiger, Phasolarctobacterium, Alstipes, Oscillibacter*, and *Sutterella*, and lower abundances of *Blautia, Anaerostipes, Clostridium* cluster XVIII, and *Streptococcus* (Rothenberg et al., 2021).

Of the studies exploring relationships between beta diversity and a composite cognitive score (consisting of motor, language and other cognitive skills), all three found significant relationships in preschoolers. One study in 3 year olds found that community structure characterized by higher abundances of *Faecalibacterium, Clostridium* cluster XIVa, *Gemmiger, Phasolarctobacterium, Alstipes, Oscillibacter*, and *Sutterella*, and lower abundances of *Blautia, Anaerostipes, Clostridium* cluster XVIII, and *Streptococcus* was associated with higher composite cognition (Rothenberg et al., 2021). Conversely, an early learning composite score (comparable to IQ) measured at age 2 years old was lowest in those with a *Faecalibacterium*-dominant community structure at 1 year old, whereas those with a *Bacteroides*-dominant community structure had the highest scores (Carlson et al., 2018). Additionally, 2 year old cognitive skills (including visual preference, attention, exploration, manipulation, concept formation) were highest in those with community structure characterized by high 1 year old abundances of Firmicutes, followed by Bacteroidetes, and lowest in those with higher Proteobacteria abundance. Community structure was also associated with change scores in cognitive skills from 1 to 2 years old, such that Firmicutes-dominant structure had the greatest improvement and Proteobacteria the lowest (Tamana et al., 2021).

Of two studies exploring relationships with personal and social skills with beta diversity, one found poorer personal and social skills at 3 years old to be associated with 3–6 months old community structure characterized by higher abundances of *Lachnospiraceae* and unclassified *Clostridiales* and negative loadings for *Bacteroides* (Sordillo et al., 2019). However, the other investigation found no associations for beta diversity and personal/social skills throughout preschool ages (Guzzardi et al., 2022).

#### 3.5.3. Taxonomy

The positive relationship of *Bifidobacterium* with cognition appears to extend to preschool ages. Indeed, *Bifidobacterium* and *Bifidobacterium longum* were positively associated with language skills at 3 years old (Zhang et al., 2021), *Bifidobacterium* (100% similarity with *B. bifidum* ATCC 29521) with fine motor skills at 18 months old (Acuna et al., 2021), and *Bifidobacteriaeceae* with fine motor skills at 3 years old (Zhang et al., 2021). Although mixed relationships with cognition were observed in infancy, *Veillonella* emerged as being primarily related to positive cognitive outcomes in older children; *Veillonella* was related to less aggressive, anxious-depressed, emotionally reactive and externalizing behaviors (Flannery et al., 2020), while *Veillonellaceae* was related to higher motor skills (Acuna et al., 2021; Zhang et al., 2021).

Other taxa emerged throughout preschool ages as having mostly positive relationships with cognition (associated with more positive and fewer negative emotions and behaviors), including *Prevotella*, *Sutterella* and *Akkermansia*. Specifically, two studies found *Sutterella* and *Prevotella* (of the Bacteroidales order) to be negatively related to total behavioral problems at 2 years old (6 months old gut microbiome measurement) (Loughman et al., 2020) and 3 years old (cross-sectionally) (Zhang et al., 2021). *Akkermansia* was also related to lower total behavior problems in preschool-aged children (Zhang et al., 2021).

Clostridia, especially *Lachnospiraceae*, were negatively related to cognition in many cases (associated with less positive and more negative emotions and behaviors). *Lachnospiraceae* was related to poorer language skills in two studies: the genus *Tyzzerella* and *Lactobacillus delbrueckii* spp. were related to poorer language skills in 3 year olds (Zhang et al., 2021). Additionally, 3–6 months old *Lachnospiraceae* was related to poorer communication skills at 3 years (Sordillo et al., 2019). Lachnospiraceae was also related to negative behaviors and emotions: higher *Lachnospiraceae* at 12 months old was related to greater behavioral problems at age 2 years, although, this was no longer significant upon adjustment for confounding variables (Loughman et al., 2020). Also a *Lachnospiraceae*, *Roseburia* was positively related to anger in another study (Sobko et al., 2020). Personal and social skills were assessed in three studies, two of which found associations between Clostridia and poor personal and social skills: *Clostridiales* order (Sordillo et al., 2019), as well as *Paraclostrium* and *Paraclostridium bifermentans* (Zhang et al., 2021).

Despite numerous other negative relationships of *Lachnospiraceae* with cognition, two studies found positive relationships for *Coprococcus* (also a *Lachnospiraceae*) with motor skills (Acuna et al., 2021; Zhang et al., 2021). Other positive relationships with cognition existed for Clostridia, such as *Eubacteriales*, including *Anaerotruncus* and *Ruminococcus* at the genus level and *Clostridium lavalense* and *Ruminococcus_spp*_N15MGS57 at the species level being positively associated with adaptive behavior (Zhang et al., 2021). Another *Eubacteriales*, *Faecalibacterium* was positively associated with motor development in one study (Rothenberg et al., 2021; Zhang et al., 2021), but negatively in another (Zhang et al., 2021).

Just as with school-aged children, *Bacteroides* demonstrated numerous, but mixed relationships with cognitive outcomes. For example, two of the five studies that measured language skills found positive associations with various *Bacteroides*, including *Bacteroides uniformis* (Tamana et al., 2021) and *Bacteroides vulgatus* (Zhang et al., 2021). *Bacteroides stercoris*, however, was negatively associated with language skills in one of these studies (Zhang et al., 2021). Interestingly, the latter study also found *Bacteroides intestinalis* and *Bacteroides vulgatus* were higher in those with more behavioral problems (Zhang et al., 2021).

Lastly, Streit et al. (2021) demonstrated unique relationships between cognitive outcomes and taxa, showing that 4 year old abundance of an unclassified genus within the family *Enterobacteriaceae*, closely resembling *Enterobacter cloacae, Enterobacter asburiae* and *Kluyyera intermedia*, were negatively associated with language skills, full-scale IQ and performance IQ. This family or species were not related to language skills in other studies.

#### 3.5.4. Metabolomics and metabolites

Volatile fatty acids were measured in one study (Loughman et al., 2020) and fecal serotonin in another in this age-group (Sobko et al., 2020). Loughman et al. (2020) found that 12 months old fecal VFAs were not related to child behavior at 2 years old (Loughman et al., 2020). The 12 weeks outdoor intervention conducted in preschool-aged children found that fecal serotonin levels were lower in the control group at post-intervention; however, while perceived stress was decreased by the intervention, there was no direct association between perceived stress and fecal serotonin (Sobko et al., 2020).

Studies used metagenomic shotgun (i.e., KEGG Orthology, Gene Ontology) (Kelsey et al., 2021; Streit et al., 2021) and 16s rRNA sequences (i.e., Phylogenetic Investigation of Communities by Reconstruction of Unobserved States (PICRUSt)) (Sobko et al., 2020) to assess and predict microbiota functional groups, respectively. In preschool aged children, higher perceived stress was positively associated with carbohydrate (i.e., pyruvate, glycolysis and gluconeogenesis), amino acid (i.e., phosphonate and phosphinate, D-Glutamine and D-glutamate, and beta-Alanine), and fatty acid metabolism. Alternatively, betalain and indole alkaloid biosynthesis were negatively associated with perceived stress (Sobko et al., 2020).

Streit et al. (2021) used a subsample of 33 children to explore metabolic potential of the gut microbiome using shotgun sequencing and found that lower cognitive functioning was associated with norspermidine synthesis at 4 years old. Tamana et al. (2021) predicted the metabolic potential at 12 months old using 16S rRNA sequencing and found no associations with cognitive outcomes at 2 years old. However, membership to the highest language and cognitive performing community structure, which was characterized by high Bacteroidetes, was associated with sphingolipid metabolism, glycosphingolipid biosynthesis and genes involved in folate, biotin, pyruvate, vitamin B6, lipoic acid, and fatty acid biosynthesis (Tamana et al., 2021).

### 3.6. Children (cognitive assessments 5 to 12 years old)

#### 3.6.1. Alpha diversity

Few studies were conducted in older children, and only one study explored gut microbiome relationships with alpha diversity: preadolescents with higher observed species had significantly higher self-reported negative emotions (Michels et al., 2019).

#### 3.6.2. Beta diversity

In 5–7 year olds, beta diversity was not associated with negative affect (Flannery et al., 2020). Similarly, an association between PCoA clusters of Weighted and Unweighted UniFrac was demonstrated with self-reported happiness subscale, but not with parent-reported emotional problems (Michels et al., 2019). One longitudinal study found the first-pass meconium composition to be related to cognition at 5 years old, after adjustment for delivery mode and sex. Specifically, meconium samples with higher abundances of *Bifidobacterium* were associated with better composite cognition, while higher abundances of *Bifidobacterium* and *Veillonella* were related to better practical reasoning abilities. Of note, there were no relationships between 3, 6, 12, or 36 months beta diversity with cognition at 6, 12, 18, 24, 36 or 60 months old (Guzzardi et al., 2022).

#### 3.6.3. Taxonomy

Relevance of taxa such as *Prevotellaceae, Veillonella, Bacteroides, Lachnospiraceae*, and *Akkermansia* to cognitive outcomes appears to extend from preschool to school-aged children, although directionality differs in some cases. For example, the positive relationships with cognition observed in preschool-aged children for *Prevotellaceae* were extended to school-age: *Prevotellaceae* UGC 001 was related to higher happiness (Michels et al., 2019). High abundances of *Akkermansia* was mixed in this age-group, showing less anxious-depressed behaviors (Flannery et al., 2020), but lower happiness (Michels et al., 2019).

Clostridia (especially *Lachnospiraceae* and *Rumminococcaceae*) demonstrated mostly negative relationships (although still mixed) with cognitive outcomes. *Rumminococcacceae* UCG 001, 002, 013, 014 and *Ruminiclostridium* 5, as well as *Lachnospiraceae* NK4A136 and FSC020 groups, *Lachnospiraceae* UCG-008, *Blautia*, *Lachnoclostridium* and *Roseburia*, were all associated with lower happiness in preadolescent children (Michels et al., 2019). *Roseburia* spp., were also positively related to anger, depressive problems, and fear in 5–7 year olds (Flannery et al., 2020). Another *Lachnospiraceae, Eubacterium*, demonstrated multiple negative relationships with cognition; *Eubacterium coprostanoligenes* group was related to lower happiness (Michels et al., 2019), while *Eubacterium rectale* and *Eubacterium siraeum* related to worse inhibitory control, a subscale of the effortful control (Flannery et al., 2020). *Eubacterium siraeum* was also positively associated with the anxious-depressed scale (Flannery et al., 2020).

Flannery et al. (2020) observed increased abundance of *Bacteroides fragilis* with higher inhibitory control ability and reduced levels of aggressive behavior, emotional reactivity, total and externalizing behavior and sadness. *Bacteroides thetaiotomicron* was also related to reduced behavior problems. *Bacteroides* was also related to greater happiness in preadolescents (Michels et al., 2019). Conversely, *Parabacteroides* (Bacteroidales order) was related to higher fear (Flannery et al., 2020).

#### 3.6.4. Metabolomics and metabolites

One study in this age-group used metagenomic shotgun (i.e., KEGG Orthology, Gene Ontology) to assess and predict microbiota functional groups, finding that fatty acid biosynthesis was positively associated with anxious depressed subscale (Flannery et al., 2020). Additionally, fear was strongly positively associated with heme biosynthesis and the manganese/iron transport system in this study. Anger and frustration was positively associated with leucine degradation, methanogenesis, glutamate transport system and GABA biosynthesis. Glutamate transport system was also positively associated with depressive problems. Impulsivity was positively associated with tryptophan metabolism to kynurenine, and anxiety problems and fear with biosynthesis of melatonin (Flannery et al., 2020). Lastly, secretion systems of type II, III, and VI, which are thought to play roles in virulence for various pathogens (Collazo and Galán, 1997; Cianciotto, 2005), were related to increased aggressive behavior, anxiety problems, anxious depression, depressive problems externalizing behavior, and anger frustration (Flannery et al., 2020).

## 4. Discussion

**FIGURE 2:**
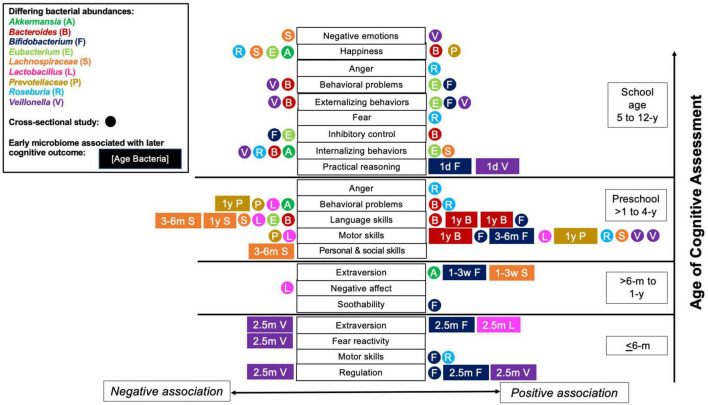
Child cognitive, temperament and Behavior outcome associations with bacterial abundances. Relationships across multiple studies between outcomes and bacterial abundances of *Bacteroides, Lachnospiraceae, Prevotellaceae, Roseburia, Bifidobacterium, Veillonella, Akkermansia*, *Eubacterium*, and *Lactobacillus* are shown (bacteria denoted by color). Each rectangle or circle symbol denotes a single study. Rectangles signify associations between bacterial abundances measured earlier in life (age of gut microbiome assessment listed inside the rectangle) and later cognitive outcomes. Circles denote cross-sectional studies. Symbols on the **left side** of x-axis (listed to the left of the cognitive outcomes) are negative associations. Symbols listed on the **right side** of the x-axis (right side of cognitive outcomes) are positive associations. The y-axis represents increasing age of cognitive assessment, from infants 6 months old and below up to school age children (5–11 years old). Y, years-of-age; m, months-of-age; w, weeks-of-age; d, days-of-age.

The present review explored the existing evidence associating gut microbial composition and/or function with the development of cognition in neurotypical children from birth to adolescence. The findings suggest a relationship between various characteristics of the gut microbiome, including community structure and the relative abundances of *Bifidobacterium, Bacteroides*, Clostridia (mainly *Lachnospiraceae*, and *Eubacterium*), *Prevotella, Veillonella, Akkermansia*, and *Roseburia* with child temperament or cognition, which is summarized in Figure 2. This figure illustrates the key genera associated with various aspects of cognition and suggests that a temporal relationship exists between microbes and cognitive outcomes. For example, *Bifidobacterium* abundance measured under 1 year old was related almost exclusively to positive cognitive outcomes. On the other hand, relationships between *Bacteroides* and *Prevotellaceae* and cognitive outcomes were not evident until 1 year old or older. *Veillonella* abundance was related to cognitive outcomes throughout all ages, whereas relationships with *Roseburia* and *Eubacterium* were primarily observed in preschool or school-aged children. These associations likely represent the developmental changes in the relative abundance of the bacteria during development, but does not preclude windows in which cognitive development may be more sensitive to different microbial consortia. This could be tested in preclinical models and human interventions to inform design and interpretation of studies within the context of the age-dependent characteristics of the gut microbiome.

Although diverse in their methodology and aims, several pilot studies of relationships between brain structural or functional connectivity and gut microbiome corroborate the numerous studies showing links between the gut microbiome and cognition (especially fear and/or negative affect), and provide potential mechanism. Integrity of functional neural circuits and regions of the brain responsible for processing and response to threat were associated with various characteristics of the gut microbiome across studies: higher alpha diversity with weaker amygdala-thalamus functional connectivity at 1 year old (Gao et al., 2019), higher alpha diversity with stronger functional connectivity of the homologous-interhemispheric network at 25 days-old (Kelsey et al., 2021), 1 month old beta diversity with 1 year old medial prefrontal cortex volume and 1 year old beta diversity with amygdala volume (Carlson et al., 2021). On the other hand, 2018 study by Carlson et al. (2018) explored associations between microbiome measures and various regional gray matter volumes, finding few consistent themes (results varied in direction, were unilateral and anatomically distributed. While promising, due to the small sample sizes, diverse methods and exploratory nature, these findings should be interpreted cautiously until replicated.

Similarly, few studies measured functional potential of the gut microbiome, yet demonstrate several consistent relationships. The significance of higher surgency or extraversion during infancy is not well-understood. High extraversion has demonstrated associations with better self-regulation and lower depressive symptoms in toddlerhood (Komsi et al., 2006), yet is related to higher externalizing behavior problems throughout childhood (e.g., aggression, anger) (Rothbart, 2007). On the other hand, stronger evidence exists for linkages between self-regulation and negative emotionality with later psychopathology in childhood and adolescence: a recent systematic review found that infants with lower self-regulation and higher negative emotionality are at increased risk for poor mental health characteristics and neurodevelopmental problems later (Kostyrka-Allchorne et al., 2020). Several studies used the same measures of alpha diversity, specifically Shannon Diversity and Chao1. These measures describe both richness and both evenness and richness, respectively (Knight et al., 2018). Neither alpha diversity measure was related to child temperament across several domains. However, those expressing greater microbiota evenness during early infancy had lower parent (Aatsinki et al., 2019) and experimenter-measured (Carlson et al., 2021) fear behavior later. Related, preschool-aged children with higher evenness demonstrated lower perceived stress (Sobko et al., 2020). Overgrowth of various bacteria with pathogenetic potential has emerged as having a potential role in cognitive and neurodevelopmental disorders (Rinninella et al., 2019). In the present review, one study measuring metabolic potential of the gut microbiome with WGS found no relationships between pathogenic virulence genes and infant temperament (Kelsey et al., 2021), while another found secretion systems relevant for virulence of pathogens associated with increased behavioral problems (Flannery et al., 2020). Higher alpha diversity and particularly evenness of the gut microbiota may contribute to the prevention of the blooming of harmful bacteria (Vangay et al., 2015) and may play a role in prevention of pathologies that impair cognitive development.

Community structure of the gut microbiome was also related to negative affect, fear and positive affect (i.e., extraversion/surgency) in most studies assessing their relationships. On the other hand, the highly heterogenous findings for the relationship between the gut microbiome and self-regulation (including aspects of inhibitory-control and effortful control) may be explained by the high dependence of self-regulation on child age (Montroy et al., 2016). Specifically, 2.5 months old community structure dominated by *Bifidobacterium/Enterobacteriaceae* exhibited higher regulating abilities at 6 months old, compared to those with *Bacteroides* or *Veillonella dispar*.-dominant community structure (Aatsinki et al., 2019). Another study found no relationship for early beta diversity with 12 months old regulation (Fox et al., 2021). In a cross-sectional study of older children (∼6 years old), taxonomic analyses revealed better inhibitory control in those with greater abundances of *Bacteroides fragilis* and lower abundances of *Eubacterium rectale, Eubacterium siraeum*, and *Bifidobacterium adolescentis* (Flannery et al., 2020). Given the inconsistency in observed relationships and the lack of replication in age groups studied, additional longitudinal studies exploring relationships between regulation and the gut microbiome are needed.

Relationships of richness, evenness and phylogenetic diversity with language and motor skills was highly mixed and essentially non-existent for higher order functions. However, Carlson et al. (2018) demonstrated that lower richness and phylogenetic diversity at 1 year old was associated with greater language development from 1 to 2 years old, even after adjusting for beta diversity and breastfeeding status at 1 year old. This finding was surprising, since alpha diversity tends to increase throughout childhood. However, consideration of dietary intake as a confounder in the alpha diversity-cognition relationship should be further explored. These findings could simply be a reflection of dietary pattern changes, as diet quality decreases throughout childhood (U.S. Department of Agriculture and U.S. Department of Health and Human Services, 2020). In other words, higher alpha diversity at 1 year old could be indicative of a child consuming a lower quality diet characteristic of an older child’s diet. Future work should carefully consider confounders in the alpha diversity-cognition relationship, including diet quality and beta diversity.

This same study by Carlson et al. (2018) along with another that replicated their analysis (Tamana et al., 2021), found that community structures characterized by elevated *Bacteroides* (Carlson et al., 2018) and Bacteroidetes (Tamana et al., 2021) were associated with better language scores at 2 years old. Additionally, they found *Bacteroides* (Carlson et al., 2018; Tamana et al., 2021) and Bacteroidetes were associated with higher change scores in language development from 1 to 2 years old. For analyses of individual taxa, two studies also found *Bacteroides* spp., *Bacteroides uniformis* (Tamana et al., 2021) and *Bacteroides vulgatus* (Zhang et al., 2021) related to higher language skills. *Bacteroides fragilis* and *Bacteroides thetaiotomicron* were also associated with fewer behavioral problems in 5–7 year olds (Flannery et al., 2020) and *Bacteroides* to greater happiness in 8–11 year olds (Michels et al., 2019). This corroborates findings in young adults, demonstrating relative abundance of *Bacteroides* associated with 20 of 33 brain-related metabolic pathways measured via prediction of functional composition of microbial communities from 16S using PICRUSt2. This study also found a relationship between *Bacteroides*-related metabolic pathways, working memory and behavioral inhibition, which was mediated by functional connectivity of the cognitive control system (prefrontal and lateral cortices) (Zhu et al., 2022).

One of the studies included in the review demonstrated that Bacteroidetes-dominant clusters in toddlers were enriched with genes related to sphingolipid metabolism (Tamana et al., 2021). Similarly, *Bacteroides* relative abundance positively associated with genes involved in sphingolipid metabolism in adults (Zhu et al., 2022). Sphingolipids serve as important structural components of cell membranes, play roles in mediation of metabolic and immune signaling within the intestines (Bryan et al., 2016) and may even be potential drivers of neurodevelopment (Hannun and Obeid, 2018). Bacteroidetes are thought to be the only commensals able to produce sphingolipids (Olsen and Jantzen, 2001). Therefore, synthesis of sphingolipids may serve as a mediator of the relationship between Bacteroidetes and cognitive development.

Two studies from this review found that children with community structures dominated by Bacteroidetes or *Bacteroides* were not only the highest cognitive performers, but that they also had increased biosynthesis of vitamins, including folate, biotin and pyridoxine (Carlson et al., 2018; Tamana et al., 2021). B vitamins are emerging as having roles in the survival and function of the gut microbiota. At the same time, certain members of the gut microbiota are thought to contribute to the host pool of B vitamins, although the extent to which this occurs is only speculative. Cobalamin, folate, niacin and pyridoxine produced by the gut microbiota may contribute between 27 and 86% of the daily recommended intakes (Magnúsdóttir et al., 2015). Cobalamin, folate and pyridoxine are particularly relevant for neurocognitive development, contributing to myelination, synaptogenesis and neurotransmitter synthesis (Reynolds, 2006; Kennedy, 2016). Interestingly, it is estimated that roughly half of all Bacteroidetes produce cobalamin, while almost all produce folate and pyridoxine (Magnúsdóttir et al., 2015).

Dominance of the *Bacteroides* genus may not be beneficial in all contexts, as it was positively associated with lower surgency and regulation in infancy (Aatsinki et al., 2019). Further, it was positively associated with higher fear reactivity in the medial prefrontal cortex in school-aged children (Callaghan et al., 2019). Zhang et al. (2021) also found that, while beneficial for language development, *Bacteroides vulgatus* was simultaneously associated with more behavioral problems. This study also reported more behavioral problems in children with higher abundances of *Bacteroides intestinalis* and *Bacteroides stercoris* (Zhang et al., 2021). Interestingly, in one study, *Bacteroides* was positively associated with amygdala volume at 1 month old-of-age (Carlson et al., 2021). Larger amygdala volumes are linked to higher anxiety-related behaviors in both adults and children (Barrós-Loscertales et al., 2006; Tottenham et al., 2010). This suggests that altered amygdala and other structures relevant to emotional processing may mediate relationships between *Bacteroides* and poor cognitive outcomes observed among these studies.

Clostridia abundance was negatively associated with language and personal/social skills, skills which are closely related with one another (Squires et al., 2009). Remarkably, these findings were replicated across two studies, in both a longitudinal and a cross-sectional context. Specifically, members of the family *Lachnospiraceae* emerged as relevant for these skills; *Tyzzerella* was related to poorer language, personal and social skills at 3 years old (Zhang et al., 2021). In early life (3–6 months old), unidentified *Lachnospiraceae*, *Lachnospiraceae dorea*, and *Lachnospira coproccus* were related to higher odds of a potential delay for communication and personal and social skills at 3 years old. Interestingly, the latter study also found that this community structure was associated with lower abundance of *Bacteroides* (Sordillo et al., 2019), which was related to greater language development during toddlerhood in several studies mentioned previously. *Lachnospiraceae* also emerged as a relevant taxa for child emotionality and behavior: higher *Lachnospiraceae* NK4A136 and FSC020 groups, *Lachnospiraceae* UCG-008, *Blautia*, *Lachnoclostridium* and *Roseburia* were related to lower happiness (Michels et al., 2019) and *Eubacterium rectale* and *siraeum* to worse inhibitory control and more reports of anxiety and depression behaviors in school-aged children (Flannery et al., 2020). Consistent with these results, *Lachnospiraceae*, including *Dorea*, *Lachnoclostridium*, and *Tyzzerella*, may be elevated in children with ASD (Ding et al., 2021; Huang et al., 2021), a disorder characterized partially by poor social communication abilities (Lord et al., 2018).

It is not surprising that higher *Bifidobacteriaeceae* abundance was related to favorable cognitive outcomes in the context of both basic functions and early child temperament, given its well-established role as conferring health benefits to the host (O’callaghan and Van Sinderen, 2016). Indeed, it appears to play roles in the treatment/prevention of colorectal cancer, diarrhea, necrotizing enterocolitis, inflammatory bowel disease and regularity of bowel movements (O’callaghan and Van Sinderen, 2016). Higher fine motor skills in toddlerhood across multiple studies were associated with bifidobacteria, including *Bifidobacteriaecae* (Zhang et al., 2021), *Bifidobacterium* (Sordillo et al., 2019; Acuna et al., 2021), and *B. bifidum* ATCC 29521 (Acuna et al., 2021). Further, the beneficial effects of supplementation with *sn*-2 palmitate-rich infant formula for 16 weeks on fine motor skills was mediated by increases in relative abundance of fecal Bifidobacteria (Wu et al., 2021). *Bifidobacterium* was also related to extraverted (Aatsinki et al., 2019; Fox et al., 2021) and regulating (Aatsinki et al., 2019; Wang et al., 2020; Kelsey et al., 2021) domains of infant temperament. The cerebellum, widely regarded as the primary supporting structure for motor skills (Evarts and Thach, 2003), also demonstrates high functional connectivity with the prefrontal and posterior parietal cortex and may therefore play roles in child behavior (Shevelkin et al., 2014). This corroborates findings in germ-free mouse studies, demonstrating capacity for *Bifidobacterium spp.* to rescue motor performance deficiencies (Luk et al., 2018) and abnormalities in synaptic density, neural activity and microglia function (Luck et al., 2020).

At the species level, two prominent commensals, *B. pseudocatenulatum* and *B. catenulatum* were positively associated with regulation (Kelsey et al., 2021). Several studies suggest an anti-inflammatory and gut barrier protective effect of these species; in a Sprague-Dawley rat model of acute liver injury, pretreatment with *B. pseudocatenulatum* LI09 and *B. catenulatum* LI10 resulted in amelioration of liver and ileal mucosal damage, as well as reduced abundance of opportunistic pathogens and bacterial translocation (Fang et al., 2017). Further, *B. pseudobatenulatum* is linked to improvements in pathologies associated with obesity (Wu et al., 2017; Sanchis-Chordà et al., 2019), lower inflammation and improvement of intestinal barrier function in a mouse colitis model (Chen et al., 2021). Bifidobacteria may support gut barrier integrity partially through its ability to produce acetate and lactate directly and butyrate indirectly, via cross-feeding mechanisms (Duncan et al., 2004). Butyrate plays important roles in the regulation of epithelial barrier integrity through stimulation of tight junction proteins (Peng et al., 2009), which may have consequences for cognitive development through its systemic effects. For example, impaired gut barrier function may result in systemic inflammation, impaired nutrient absorption, and immune homeostasis, all of which may have negative consequences for cognitive health (Shields et al., 2017; Syme et al., 2019; Blaak et al., 2020; Tommaso et al., 2021; Ren et al., 2022).

Several studies found *Prevotellaceae* to be related to positive cognitive outcomes, including fewer behavioral problems in toddlers (Loughman et al., 2020; Zhang et al., 2021) and happiness in school-aged children (Michels et al., 2019). These findings are consistent with observations in adults; higher relative abundance of Prevotella has been linked to increases in functional connectivity of the default mode network, the cognitive control network, and the fronto-parietal attention network in females (Kohn et al., 2021). The hypothalamic-pituitary-axis (HPA) is responsible for regulation of stress responses, and is therefore also relevant for behavioral problems and emotional reactivity (Doom and Gunnar, 2013). Dysregulation of the HPA is evident in germ-free mice (Clarke et al., 2013), and higher *Prevotella* abundance has been linked to lower HPA reactivity in infants, measured via salivary cortisol before and after heel stick (Rosin et al., 2021). Abundance of *Prevotella* is widely regarded as increasing as a result of consumption of plant-derived polysaccharides in children (Dinsmoor et al., 2021), a covariate that was only accounted for in 1/3 studies demonstrating these associations. Future studies should aim to account for consumption of dietary fiber and protein (both fermentable by the gut commensals), in order to understand if *Prevotella* relates to cognitive development, independent of diet.

Studies in germ-free mice suggest a role of SCFAs in the rescue of structural and immune-specific abnormalities (Erny et al., 2015), potentially due to their systemic anti-inflammatory effects (Ren et al., 2022). Tamana et al. (2021) found that children with higher 1 year old fatty acid biosynthesis and Bacteroidetes abundance had significantly better cognitive and language performance at age two. However, functional potential for fatty acid metabolism in the gut microbiome was also positively related to anxiety (Flannery et al., 2020) and perceived stress (Sobko et al., 2020) in school- and preschool-aged children, respectively. Functional terms for fatty acid metabolism does not differentiate between production of SCFAs versus BCFAs. Further, BCFAs are related to poor outcomes for gut and metabolic health (Russell et al., 2011), both of which have consequences for cognitive health (although this has yet to be directly explored). BCFAs are produced in greater abundance in the distal colon from fermentation of proteins (Macfarlane et al., 1992); therefore, higher BCFAs may be indirectly representative of low fiber intake, since, in the absence of this preferred source, microbes may switch to fermentation of proteins (François et al., 2014; Hald et al., 2016). Indeed, elevated branched-chain fatty acids (BCFAs) were associated with more emotional problems in older children (8–16 years), independently of BMI and diet (Michels et al., 2017).

Butyrate is widely regarded as having positive benefits for the host (Blaak et al., 2020). However, the study mentioned previously also found butyrate to be associated with more emotional problems (Michels et al., 2017). While this could indicate previous overestimation of these positive effects of butyrate, or that butyrate is specifically detrimental to child cognitive health, measurement of VFAs in feces is significantly limited in its ability to differentiate between increased production versus low absorption by enterocytes. Thus, elevated excretion of butyrate could be a product of impaired absorption of SCFAs. Interestingly, Michels et al. (2017) did not observe associations between other SCFAs, propionate or acetate, wherein acetate and lactate (a SCFA intermediate) are frequently converted to butyrate by gut commensals (Duncan et al., 2004; O’callaghan and Van Sinderen, 2016). Given the complexity of interpretation of fecal VFA or related functional genes alone, studies that incorporate both metabolites and functional capacity of the gut microbiome simultaneously are needed in younger cohorts.

The tryptophan/serotonin/kynurenine pathway has recently emerged as an important contributor to the gut-microbiome-brain axis through its modulation of ENS and CNS functioning. Specifically, the gut microbiota appear to directly influence host serotonergic production through alteration of tryptophan availability via its metabolism (Kennedy, 2016), as well as through microbial metabolites such as SCFAs (Reigstad et al., 2015). One study included in the present review found that a 12 weeks outdoor intervention trial reduced perceived stress in preschool-aged children. Fecal serotonin remained stable and gut microbial composition was altered in the intervention group, but not in the control group. *Roseburia spp*. were negatively associated with fecal serotonin and positively associated with anger frequency, providing evidence for altered behavior through regulation of circulating tryptophan availability and kynurenine pathway metabolism by the gut microbiota (Sobko et al., 2020). The role of the gut microbiota in immune system development may also indirectly influence this pathway, as the trade-off between production of kynurenine and serotonin favors production of kynurenine in times of stress and inflammation (Kennedy, 2016). In fact, another study in similarly, aged children demonstrated positive associations for impulsivity and adverse home environment exposure (likely to impose stress upon a child) with tryptophan metabolism to kynurenine by the gut microbiome. Further, impulsivity was linked to lower abundances of *Bacteroides fragilis* and higher abundances of *Eubacterium siraeum*. Consistent with the outdoor intervention study outlined above, Flannery et al. (2020) also found higher *Roseburia hominis* to be linked to an increased anger-frustration subscale.

Given the limited evidence on gut microbiome-cognition relationships in neurotypical children, a wide range of neurocognitive outcomes were included in the present review. Thus, one of the prominent limiting factors for summarizing findings was the lack of replication of methodological approaches for neurocognitive assessment. As a result, there was little uniformity between studies for cognitive outcome and age. This is a significant limitation, as both the human gut microbiome (Bokulich et al., 2016; Stewart et al., 2018) and cognitive functions (Casey et al., 2005) develop rapidly throughout early life. Future work should aim to replicate findings of high quality studies reviewed herein.

As seen in Supplementary Table 1, the most common form of neurocognitive assessments were child behavioral problems, infant temperament characteristics, and motor and language skills. Far less work has focused on independent cognitive domains (i.e., working memory or inhibitory control), which comprise cognitive control, or “the ability to flexibly adjust behavior in the context of dynamically changing goals and task demands” (Nigg, 2017). This aspect of cognition provides a foundation for problem solving, reasoning and planning (Diamond, 2013), and is relevant for later academic achievement (Blair and Razza, 2007), physical health (Moffitt et al., 2011), and mental health (Tangney et al., 2004). Conversely, while there is some evidence suggesting negative emotionality as a predictor of later psychological disorder, relevance of other domains (regulation/orienting and surgency/extraversion) to child health are less clear (Kostyrka-Allchorne et al., 2020). Related, the present study attempted to include “neurotypical” infants only, while, neurodevelopmental disorders are often undetected throughout infancy (Straub et al., 2022). As a result, studies on infant temperament may be biased by inclusion of undiagnosed neurodevelopmental conditions. Thus, studies focused on cognitive control or its domains are needed to complement current work comparing thosewithout neurodevelopmental disorders to fully understand applicability of interventions aimed at the gut microbiome to promote cognitive health.

Another finding of this review was the lack of gut microbiome-cognition-axis studies conducted in older children, especially school-aged. Research should certainly address this area, as the gut microbiome is associated with mood disorders (Valles-Colomer et al., 2019), which are commonly incident and increasing in prevalence for this age-group (Merikangas et al., 2009). Further, development of many cognitive processes and related structures continues to occur throughout late-childhood and even early adulthood (Casey et al., 2005). For example, the prefrontal cortex, which contributes to our self-regulating abilities, develops until early adulthood (Nigg, 2017). Interestingly, a small pilot study of children ∼8 years old observed that higher abundances of *Lachnospiraceae* and *Bacteroides* were related to elevated prefrontal cortex activation to emotional faces (Callaghan et al., 2019). The relevance of the gut microbiome for mood-disorders is further supported by its observed relationships with mood-disorder-related symptomology and behaviors, as higher alpha diversity and adundances of *Bacteroides* and *Parabacteroides*, yet lower abundance of *Lachnospiracae* and *Veillonella* was related to at least one measure of elevated stress (i.e., parent-reported negative events and emotions, child-reported emotional problems and low happiness, parasympathetic response to stress), independent of age, gender, parental education, BMI z-score, fiber, protein, sweet and fat food intake, physical activity and sleep (Michels et al., 2019).

Many studies included in the present review were cross-sectional, thus lacking study design to infer causality of the gut microbiome on cognitive development. Species-level observations herein should be interpreted cautiously, as most studies (*n* = 20/23) measured gut microbiota via 16S rRNA, and thus had limited capacity to infer species-level relationships (Knight et al., 2018). There were also considerable differences in sequencing techniques, metagenomic analyses and bioinformatic analyses. For example, 16S rRNA regions, qPCR primers, sequencing platforms and reference databases varied, all of which introduce biases related to sequencing depth and coverage, as well as identification of microbes and related genes (Knight et al., 2018).

Most studies were conducted in children under age 3 years old, wherein the gut microbiome is thought to be highly unstable and variable up to this point (Stewart et al., 2018). Additionally, cognitive outcomes prior to the start of formal schooling are likely to be influenced heavily by parent-child relationships and home or daycare environments, all of which can also impact gut microbiome colonization (Tamburini et al., 2016; Stewart et al., 2018). In fact, one study from the present review observed a moderating effect of caregiver behavior on the relationships between the gut microbiome functional diversity and behavioral dysregulation (Flannery et al., 2020). By conducting longitudinal analyses that include gut microbiome measurement at multiple timepoints before and after gut microbiome stability increases and cognitive assessments after the initiation of formal schooling, several confounders of the gut microbiome-cognition relationship could be avoided.

Several covariates considered in these studies may require additional consideration for future gut microbiome-cognition studies. Certain aspects of human brain and cognitive development may be dependent on sex (Hastings et al., 2011), and preclinical evidence suggests that boys may be more susceptible to gut microbiome effects on cognitive development (Turnbaugh et al., 2007, Jasarevic et al., 2017). While all studies included in this review accounted for sex as a covariate at least statistically, several explored relationships between cognitive outcomes and gut microbiome separately and observed sex-dependent relationships (Christian et al., 2015; Aatsinki et al., 2019; Tamana et al., 2021). Future studies should aim to explore the gut microbiome-cognition axis together, as well as separately for males and females. Studies with gut microbiome measured post-weaning did not account for solid diet as a covariate, which is highly influential for gut microbiome colonization/development (Davis et al., 2020; Dinsmoor et al., 2021). Also, a remarkable number of studies (*n* = 5) did not adjust for antibiotic exposure. Due to the significant impact antibiotic usage can have on development of the gut microbiome (Bokulich et al., 2016), this may represent an important confounder for the gut microbiome-cognition/behavior relationship. Lastly, assessment of the included studies found that 15/23 of these to be of “fair” and two to be of “poor” quality, suggesting that results of many studies included in the review are subject to at least some bias.

## 5. Conclusion

Research into potential associations between the gut microbiota and cognitive development in neurotypical children has lagged behind studies of children with atypical neurodevelopmental trajectories. However, existing evidence support microbial family and genus-level relationships with favorable cognitive outcomes in children. Although, it should be noted that specific microbial taxa can have both positive and negative associations with cognitive outcomes in children of different ages. To advance the field, future studies should explore microbial associations at the species or strain-level gut microbiota and should incorporate metagenomic and metabolomic analyses to uncover functional readouts of the microbiome. In addition, dietary intakes should be routinely collected given its key role in modulating gut microbiome-diet interactions in infants and children (Davis et al., 2020; Dinsmoor et al., 2021). Additionally, evidence-informed RCT dietary interventions represent a promising avenue by which to engineer the gut microbiome, given its feasibility in both clinical and non-clinical settings. Although this work was focused on healthy and neurotypically developing children, studies included in this review provide a promising foundation for future gut-microbiome-targeted work aimed at alleviating neurodevelopment delays throughout childhood.

## Author contributions

AM, SD, and NK: design of search strategy. AM and MA-L: conducted study selections, data collection, and quality assessments. MA-L, CC, NK, and SD: critical reading of the manuscript. AM: drafting and refining of the manuscript. All authors contributed to the article and approved the submitted version.

## References

[B1] Aatsinki A.-K. Kataja E.-L. Munukka E. Lahti L. Keskitalo A. Korja R. (2020). Infant fecal microbiota composition and attention to emotional faces. *Emotion* 2020:924. 10.1037/EMO0000924 33382324

[B2] Aatsinki A.-K. Lahti L. Uusitupa H.-M. Munukka E. Keskitalo A. Nolvi S. (2019). Gut microbiota composition is associated with temperament traits in infants. *Brain. Behav. Immun.* 80 849–858. 10.1016/j.bbi.2019.05.035 31132457

[B3] Achenbach T. M. (1999). “The child behavior checklist and related instruments,” in *The use of psychological testing for treatment planning and outcomes assessment*, ed. Maruish M. E. (Lawrence Erlbaum Associates Publishers), 429–466.

[B4] Acuna I. Cerdo T. Ruiz A. Torres-Espinola F. J. Lopez-Moreno A. Aguilera M. (2021). Infant gut microbiota associated with fine motor skills. *Nutrients* 13:1673. 10.3390/nu13051673 34069166 PMC8156744

[B5] Baldi S. Mundula T. Nannini G. Amedei A. (2021). World journal of gastroenterology microbiota shaping-the effects of probiotics, prebiotics, and fecal microbiota transplant on cognitive functions: a systematic review conflict-of-interest statement: PRISMA 2009 checklist statement. *World J. Gastroenterol.* 27 6715–6732. 10.3748/wjg.v27.i39.6715 34754163 PMC8554405

[B6] Barrós-Loscertales A. Meseguer V. Sanjuán A. Belloch V. Parcet M. A. Torrubia R. (2006). Behavioral inhibition system activity is associated with increased amygdala and hippocampal gray matter volume: a voxel-based morphometry study. *NeuroImage* 33 1011–1015. 10.1016/J.NEUROIMAGE.2006.07.025 16979909

[B7] Bayley N. (1993). *Bayley scales of infant and toddler development.* San Antonio, TX: Psychological Corporation.

[B8] Bayley N. (2006). *Bayley scales of infant development.* San Antonio, TX: The Psychological Corporation.

[B9] Blaak E. E. Canfora E. E. Theis S. Frost G. Groen A. K. Mithieux G. (2020). Short chain fatty acids in human gut and metabolic health. *Sensus R. Cosun* 11 411–455. 10.3920/BM2020.0057 32865024

[B10] Blair C. Razza R. P. (2007). Relating effortful control, executive function, and false belief understanding to emerging math and literacy ability in kindergarten. *Child Dev.* 78 647–663. 10.1111/j.1467-8624.2007.01019.x 17381795

[B11] Bokulich N. A. Chung J. Battaglia T. Henderson N. Jay M. Li H. (2016). Antibiotics, birth mode, and diet shape microbiome maturation during early life. *Sci. Trans. Med.* 2016:7121. 10.1126/scitranslmed.aad7121 27306664 PMC5308924

[B12] Borre Y. E. O’keeffe G. W. Clarke G. Stanton C. Dinan T. G. Cryan J. F. (2014). Microbiota and neurodevelopmental windows: implications for brain disorders. *Trends Mol. Med.* 20 509–518. 10.1016/j.molmed.2014.05.002 24956966

[B13] Bryan P.-F. Karla C. Edgar Alejandro M.-T. Sara Elva E.-P. Gemma F. Luz C. (2016). Sphingolipids as mediators in the crosstalk between microbiota and intestinal cells: implications for inflammatory bowel disease. *Med. Inflamm.* 2016:9890141. 10.1155/2016/9890141 27656050 PMC5021499

[B14] Bundgaard-Nielsen C. Knudsen J. Leutscher P. D. C. Lauritsen M. B. Nyegaard M. Hagstrøm S. (2020). Gut microbiota profiles of autism spectrum disorder and attention deficit/hyperactivity disorder: a systematic literature review. *Gut Microbes.* 11 1172–1187. 10.1080/19490976.2020.1748258 32329656 PMC7524304

[B15] Callaghan B. L. Fields A. Gee D. G. Gabard-Durnam L. Caldera C. Humphreys K. L. (2019). Mind and gut: associations between mood and gastrointestinal distress in children exposed to adversity. *Dev. Psychopathol.* 32:87. 10.1017/S0954579419000087 30919798 PMC6765443

[B16] Carlson A. L. Xia K. Azcarate-Peril M. A. Goldman B. D. Ahn M. Styner M. A. (2018). Infant gut microbiome associated with cognitive development. *Biol. Psychiatry* 83 148–159. 10.1016/j.biopsych.2017.06.021 28793975 PMC5724966

[B17] Carlson A. L. Xia K. Azcarate-Peril M. A. Rosin S. P. Fine J. P. Mu W. (2021). Infant gut microbiome composition is associated with non-social fear behavior in a pilot study. *Nat. Commun.* 12 281. 10.1038/s41467-021-23281-y 34078892 PMC8172562

[B18] Casey B. J. Tottenham N. Liston C. Durston S. (2005). Imaging the developing brain: what have we learned about cognitive development? *Trends Cogn. Sci.* 9 104–110. 10.1016/J.TICS.2005.01.011 15737818

[B19] Chen Y. Yang B. Stanton C. Ross R. P. Zhao J. Zhang H. (2021). Bifidobacterium pseudocatenulatum ameliorates DSS-induced colitis by maintaining intestinal mechanical barrier, blocking proinflammatory cytokines, inhibiting TLR4/NF-κB signaling, and altering gut microbiota. *J. Agric. Food Chem.* 69 1496–1512. 10.1021/acs.jafc.0c06329 33512996

[B20] Christian L. M. Galley J. D. Hade E. M. Schoppe-Sullivan S. Dush C. K. Bailey M. T. (2015). Gut microbiome composition is associated with temperament during early childhood. *Brain. Behav. Immun.* 45 118–127. 10.1016/j.bbi.2014.10.018 25449582 PMC4342262

[B21] Cianciotto N. P. (2005). Type II secretion: a protein secretion system for all seasons. *Trends Microbiol.* 13 581–588. 10.1016/J.TIM.2005.09.005 16216510

[B22] Clarke G. Grenham S. Scully P. Fitzgerald P. Moloney R. D. Shanahan F. (2013). The microbiome-gut-brain axis during early life regulates the hippocampal serotonergic system in a sex-dependent manner. *Mol. Psychiatry* 18 666–673. 10.1038/mp.2012.77 22688187

[B23] Collazo C. M. Galán J. E. (1997). The invasion-associated type-III protein secretion system in *Salmonella* – a review. *Gene* 192 51–59. 10.1016/S0378-1119(96)00825-6 9224874

[B24] Davis E. C. Dinsmoor A. M. Wang M. Donovan S. M. (2020). Microbiome composition in pediatric populations from birth to adolescence: impact of diet and prebiotic and probiotic interventions. *Dig. Dis. Sci.* 65 706–722. 10.1007/s10620-020-06092-x 32002758 PMC7046124

[B25] Diamond A. (2013). Executive functions. *Annu. Rev. Psychol.* 64 135–168. 10.1146/annurev-psych-113011-143750 23020641 PMC4084861

[B26] Ding H. Yi X. Zhang X. Wang H. Liu H. Mou W.-W. (2021). Imbalance in the gut microbiota of children with. *Autism Spectr. Disord. Front. Cell Infect. Microbiol.* 11:572752. 10.3389/fcimb.2021.572752 34790583 PMC8591234

[B27] Dinsmoor A. M. Aguilar-Lopez M. Khan N. A. Donovan S. M. (2021). A systematic review of dietary influences on fecal microbiota composition and function among healthy humans 1–20 years of age. *Adv. Nutr.* 12 1734–1750. 10.1093/ADVANCES/NMAB047 33951139 PMC8483965

[B28] Doom J. R. Gunnar M. R. (2013). Stress physiology and developmental psychopathology: past, present, and future. *Dev. Psychopathol.* 25 1359–1373. 10.1017/S0954579413000667 24342845 PMC3869040

[B29] Duncan S. H. Louis P. Flint H. J. (2004). Lactate-utilizing bacteria, isolated from human feces, that produce butyrate as a major fermentation product. *Appl. Environ. Microbiol.* 70 5810–5817. 10.1128/AEM.70.10.5810-5817.2004/FORMAT/EPUB 15466518 PMC522113

[B30] Erny D. Angelis A. L. H. Jaitin D. Wieghofer P. Staszewski O. David E. (2015). Host microbiota constantly control maturation and function of microglia in the CNS. *Nat. Neurosci.* 18:965. 10.1038/NN.4030 26030851 PMC5528863

[B31] Evarts E. V. Thach W. T. (2003). Motor mechanisms of the CNS: cerebrocerebellar interrelations. *Annu Rev. Physiol.* 31 451–498. 10.1146/ANNUREV.PH.31.030169.002315 4885774

[B32] Fang D. Shi D. Lv L. Gu S. Wu W. Chen Y. (2017). *Bifidobacterium pseudocatenulatum* LI09 and *Bifidobacterium catenulatum* LI10 attenuate D-galactosamine-induced liver injury by modifying the gut microbiota. *Sci. Rep.* 7:8770. 10.1038/s41598-017-09395-8 28821814 PMC5562910

[B33] Flannery J. E. Stagaman K. Burns A. R. Hickey R. J. Roos L. E. Giuliano R. J. (2020). Gut Feelings begin in childhood: the gut metagenome correlates with early environment, caregiving, and behavior. *MBIO* 11:2780. 10.1128/mBio.02780-19 31964729 PMC6974564

[B34] Fox M. Lee S. M. Wiley K. S. Lagishetty V. Sandman C. A. Jacobs J. P. (2021). Development of the infant gut microbiome predicts temperament across the first year of life. *Dev. Psychopathol.* 2021:456. 10.1017/S0954579421000456 34108055 PMC9463039

[B35] François I. E. J. A. Lescroart O. Veraverbeke W. S. Marzorati M. Possemiers S. Hamer H. (2014). Effects of wheat bran extract containing arabinoxylan oligosaccharides on gastrointestinal parameters in healthy preadolescent children. *J. Pediatr. Gastroenterol. Nutr.* 58 647–653. 10.1097/MPG.0000000000000285 24368315

[B36] Gao W. Salzwedel A. P. Carlson A. L. Xia K. Andrea A. P. Styner M. A. (2019). Gut microbiome and brain functional connectivity in infants-a preliminary study focusing on the amygdala. *Psychopharmacology* 2019:5161. 10.1007/s00213-018-5161-8 30604186 PMC6599471

[B37] Gao X. Huynh B.-T. Guillemot D. Glaser P. Opatowski L. (2018). Inference of significant microbial interactions from longitudinal metagenomics data. *Front. Microbiol.* 9:2319. 10.3389/fmicb.2018.02319 30386306 PMC6198172

[B38] Guzzardi M. A. Ederveen T. H. A. Rizzo F. Weisz A. Collado M. C. Muratori F. (2022). Maternal pre-pregnancy overweight and neonatal gut bacterial colonization are associated with cognitive development and gut microbiota composition in pre-school-age offspring. *Brain. Behav. Immun.* 100 311–320. 10.1016/J.BBI.2021.12.009 34920092

[B39] Hald S. Grethe Schioldan A. Moore M. E. Dige A. Nygaard Laerke H. Agnholt J. (2016). Effects of arabinoxylan and resistant starch on intestinal microbiota and short-chain fatty acids in subjects with metabolic syndrome: a randomised crossover study. *PLoS One* 11:e0159223. 10.1371/journal.pone.0159223 27434092 PMC4951149

[B40] Hannun Y. A. Obeid L. M. (2018). Sphingolipids and their metabolism in physiology and disease. *Nat. Rev. Mol. Cell Biol.* 19 175–191. 10.1038/nrm.2017.107 29165427 PMC5902181

[B41] Hastings P. D. Ruttle P. L. Serbin L. A. Mills R. S. L. Stack D. M. Schwartzman A. E. (2011). Adrenocortical responses to strangers in preschoolers: relations with parenting, temperament, and psychopathology. *Dev. Psychobiol.* 53 694–710. 10.1002/DEV.20545 21432849

[B42] Higgins J. Altman D. Gøtzsche P. Jüni P. Moher D. Oxman A. (2011). The cochrane collaboration’s tool for assessing risk of bias in randomised trials. *BMJ* 343:d5928. 10.1136/bmj.d5928 22008217 PMC3196245

[B43] Huang M. Liu J. Liu K. Chen J. Wei Z. Feng Z. (2021). Microbiome-specific statistical modeling identifies interplay between gastrointestinal microbiome and neurobehavioral outcomes in patients with autism: a case control study. *Front. Psychiatry* 12:682454. 10.3389/FPSYT.2021.682454/FULL PMC8563626 34744810

[B44] Iglesias-Vázquez L. Van G. Riba G. Arija V. Canals J. (2020). Composition of gut microbiota in children with autism spectrum disorder: a systematic review and meta-analysis. *Nutrients* 12:792. 10.3390/nu12030792 32192218 PMC7146354

[B45] Jasarevic E. Howard C. D. Misic A. M. Beiting D. P. Bale T. L. (2017). Stress during pregnancy alters temporal and spatial dynamics of the maternal and offspring microbiome in a sex-specific manner. *Sci. Rep.* 7:44182. 10.1038/srep44182 28266645 PMC5339804

[B46] Kelsey C. M. Prescott S. McCulloch J. A. Trinchieri G. Valladares T. L. Dreisbach C. (2021). Gut microbiota composition is associated with newborn functional brain connectivity and behavioral temperament. *Brain. Behav. Immun.* 91 472–486. 10.1016/j.bbi.2020.11.003 33157257

[B47] Kennedy D. O. (2016). B vitamins and the brain: mechanisms, dose and efficacy-a review. *Nutrients* 8:68. 10.3390/nu8020068 26828517 PMC4772032

[B48] Knight R. Vrbanac A. Taylor B. C. Aksenov A. Callewaert C. Debelius J. (2018). Best practices for analysing microbiomes. *Nat. Rev. Microbiol.* 16 410–422. 10.1038/s41579-018-0029-9 29795328

[B49] Kohn N. Szopinska-Tokov J. Arenas A. L. Beckmann C. F. Arias-Vasquez A. Aarts E. (2021). Multivariate associative patterns between the gut microbiota and large-scale brain network connectivity. *Gut Microbes* 13:2006586. 10.1080/19490976.2021.2006586 34856861 PMC8726725

[B50] Komsi N. Räikkönen K. Pesonen A. K. Heinonen K. Keskivaara P. Järvenpää A. L. (2006). Continuity of temperament from infancy to middle childhood. *Infant Behav. Dev.* 29 494–508. 10.1016/J.INFBEH.2006.05.002 17138302

[B51] Kostyrka-Allchorne K. Wass S. V. Sonuga-Barke E. J. S. (2020). Research review: do parent ratings of infant negative emotionality and self-regulation predict psychopathology in childhood and adolescence? A systematic review and meta-analysis of prospective longitudinal studies. *J. Child Psychol. Psychiatry* 61 401–416. 10.1111/JCPP.13144 31696514

[B52] Lord C. Elsabbagh M. Baird G. Veenstra-Vanderweele J. (2018). Autism spectrum disorder. *Lancet* 2018:31129. 10.1016/S0140-6736(18)31129-2 30078460 PMC7398158

[B53] Loughman A. Ponsonby A.-L. O’Hely M. Symeonides C. Collier F. Tang M. L. K. (2020). Gut microbiota composition during infancy and subsequent behavioural outcomes. *Ebiomedicine* 52:102640. 10.1016/j.ebiom.2020.102640 32062351 PMC7016366

[B54] Luck B. Engevik M. A. Ganesh B. P. Lackey E. P. Lin T. Balderas M. (2020). Bifidobacteria shape host neural circuits during postnatal development by promoting synapse formation and microglial function. *Sci. Rep.* 10:64173. 10.1038/s41598-020-64173-3 32385412 PMC7210968

[B55] Luk B. Veeraragavan S. Engevik M. Balderas M. Major A. Runge J. (2018). Postnatal colonization with human “infant-type” bifidobacterium species alters behavior of adult gnotobiotic mice. *PLoS One* 13:510. 10.1371/JOURNAL.PONE.0196510 29763437 PMC5953436

[B56] Macfarlane G. T. Gibson G. R. Beatty E. Cummings J. H. (1992). Estimation of short-chain fatty acid production from protein by human intestinal bacteria based on branched-chain fatty acid measurements. *FEMS Microbiol. Ecol.* 101 81–88. 10.1111/j.1574-6968.1992.tb05764.x

[B57] Magnúsdóttir S. Ravcheev D. De Crécy-Lagard V. Thiele I. (2015). Systematic genome assessment of B-vitamin biosynthesis suggests co-operation among gut microbes. *Front. Genet.* 6:148. 10.3389/FGENE.2015.00148 25941533 PMC4403557

[B58] Merikangas K. R. Nakamura E. F. Kessler R. C. (2009). Epidemiology of mental disorders in children and adolescents. *Dialogues Clin. Neurosci.* 11 7–20. 10.31887/DCNS.2009.11.1/krmerikangas 19432384 PMC2807642

[B59] Michels N. Van de Wiele T. De Henauw S. (2017). Chronic psychosocial stress and gut health in children. *Psychosom. Med.* 79 927–935. 10.1097/PSY.0000000000000413 27787408

[B60] Michels N. Van de Wiele T. Fouhy F. O’Mahony S. Clarke G. Keane J. (2019). Gut microbiome patterns depending on children’s psychosocial stress: reports versus biomarkers. *Brain. Behav. Immun.* 80 751–762. 10.1016/J.BBI.2019.05.024 31112792

[B61] Moffitt T. E. Arseneault L. Belsky D. Dickson N. Hancox R. J. Harrington H. (2011). A gradient of childhood self-control predicts health, wealth, and public safety. *Soc. Sci.* 108 2693–2698. 10.1073/pnas.1010076108 21262822 PMC3041102

[B62] Moher D. Shamseer L. Clarke M. Ghersi D. Liberati A. Petticrew M. (2016). Preferred reporting items for systematic review and meta-analysis protocols (PRISMA-P) 2015 statement. *Rev. Espanola Nutr. Humana Diet.* 20 148–160. 10.1186/2046-4053-4-1/TABLES/4 PMC4320440 25554246

[B63] Montroy J. J. Bowles R. P. Skibbe L. E. McClelland M. M. Morrison Janelle J. Montroy F. J. (2016). The development of self-regulation across early childhood. *Dev. Psychol.* 2016:159. 10.1037/dev0000159 27709999 PMC5123795

[B64] Mullen E. (1995). *Mullen scales of early development*. Circle Pines, MN: American Guidance Service, Inc.

[B65] Nigg J. T. (2017). Annual research review: on the relations among self-regulation, self-control, executive functioning, effortful control, cognitive control, impulsivity, risk-taking, and inhibition for developmental psychopathology. *J. Child Psychol. Psychiatry* 58 361–383. 10.1111/JCPP.12675 28035675 PMC5367959

[B66] O’callaghan A. Van Sinderen D. (2016). Bifidobacteria and their role as members of the human gut microbiota. *Front. Microbiol. Wwwfrontiersinorg* 1:925. 10.3389/fmicb.2016.00925 27379055 PMC4908950

[B67] Olsen I. Jantzen E. (2001). Sphingolipids in bacteria and fungi. *Anaerobe* 7 103–112. 10.1006/ANAE.2001.0376

[B68] Peng L. Li Z.-R. Green R. S. Holzman I. R. Lin J. (2009). Butyrate enhances the intestinal barrier by facilitating tight junction assembly via activation of AMP-activated protein kinase in Caco-2 cell monolayers 1,2. *J. Nutr. Biochem. Mol. Genet. Mech.* 139 1619–1625. 10.3945/jn.109.104638 19625695 PMC2728689

[B69] Putnam S. P. Helbig A. L. Gartstein M. A. Rothbart M. K. Leerkes E. (2014). Development and assessment of short and very short forms of the infant behavior questionnaire–revised. *J. Pers. Assess.* 96 445–458. 10.1080/00223891.2013.841171 24206185

[B70] Reigstad C. S. Salmonson C. E. Rainey J. F. Szurszewski J. H. Linden D. R. Sonnenburg J. L. (2015). Gut microbes promote colonic serotonin production through an effect of short-chain fatty acids on enterochromaffin cells. *FASEB J.* 29 1395–1403. 10.1096/FJ.14-259598 25550456 PMC4396604

[B71] Ren M. Li H. Fu Z. Li Q. (2022). Centenarian-sourced lactobacillus casei combined with dietary fiber complex ameliorates brain and gut function in aged mice. *Nutrients* 14:324. 10.3390/nu14020324 35057509 PMC8781173

[B72] Reynolds E. (2006). Vitamin B12, folic acid, and the nervous system. *Lancet Neurol.* 5 949–960. 10.1016/S1474-4422(06)70598-1 17052662

[B73] Rinninella E. Raoul P. Cintoni M. Franceschi F. Miggiano G. A. D. Gasbarrini A. (2019). What is the healthy gut microbiota composition? A changing ecosystem across age, environment, diet, and diseases. *Microorganisms* 7:10014. 10.3390/MICROORGANISMS7010014 30634578 PMC6351938

[B74] Rosin S. Xia K. Azcarate-Peril M. A. Carlson A. L. Propper C. B. Thompson A. L. (2021). A preliminary study of gut microbiome variation and HPA axis reactivity in healthy infants. *Psychoneuroendocrinology* 124:105046. 10.1016/j.psyneuen.2020.105046 33254059 PMC8121098

[B75] Rothbart M. K. (2007). Temperament, development, and personality. *Curr. Dir. Psychol. Sci*. 16, 207–212. 10.1111/j.1467-8721.2007.00505.x

[B76] Rothbart M. K. Ellis L. K. Posner M. I. (2004). “Temperament and self-regulation,” in *Handbook of self-regulation: research, theory, and applications*, eds Baumeister K. D. Roy F. (New York: Guilford Press), 357–370.

[B77] Rothenberg S. E. Chen Q. Shen J. Nong Y. Nong H. Trinh E. P. (2021). Neurodevelopment correlates with gut microbiota in a cross-sectional analysis of children at 3 years of age in rural China. *Sci. Rep.* 11:86761. 10.1038/s41598-021-86761-7 33795717 PMC8016964

[B78] Russell W. R. Gratz S. W. Duncan S. H. Holtrop G. Ince J. Scobbie L. (2011). High-protein, reduced-carbohydrate weight-loss diets promote metabolite profiles likely to be detrimental to colonic health. *Am. J. Clin. Nutr.* 93 1062–1072. 10.3945/AJCN.110.002188 21389180

[B79] Sanchis-Chordà J. Gómez Del Pulgar E. M. Carrasco-Luna J. Sanz Y. Codoñer-Franch P. (2019). *Bifidobacterium pseudocatenulatum* CECT 7765 supplementation improves inflammatory status in insulin-resistant obese children. *Eur. J. Nutr.* 58 2789–2800. 10.1007/s00394-018-1828-5 30251018

[B80] Sela T. Lavidor M. (2014). “Chapter 11 - high-level cognitive functions in healthy subjects,” in *The stimulated brain*, ed. Kadosh R. C. (San Diego: Academic Press), 299–329. 10.1016/B978-0-12-404704-4.00011-9

[B81] Shevelkin A. V. Ihenatu C. Pletnikov M. V. (2014). Pre-clinical models of neurodevelopmental disorders: focus on the cerebellum. *Rev. Neurosci.* 2014:49. 10.1515/revneuro-2013-0049 24523305 PMC4052755

[B82] Shields G. S. Moons W. G. Slavich G. M. (2017). Inflammation, self-regulation, and health: an immunologic model of self-regulatory failure. *Perspect. Psychol. Sci.* 12 588–612. 10.1177/1745691616689091 28679069 PMC5519413

[B83] Sobko T. Liang S. Cheng W. H. G. Tun H. M. (2020). Impact of outdoor nature-related activities on gut microbiota, fecal serotonin, and perceived stress in preschool children: the Play&Grow randomized controlled trial. *Sci. Rep.* 10:78642. 10.1038/s41598-020-78642-2 33319792 PMC7738543

[B84] Sobko T. Tse M. Kaplan M. (2016). A randomized controlled trial for families with preschool children-promoting healthy eating and active playtime by connecting to nature. *BMC Public Health* 16:505. 10.1186/s12889-016-3111-0 27296723 PMC4906726

[B85] Sordillo J. E. Korrick S. Laranjo N. Carey V. Weinstock G. M. Gold D. R. (2019). Association of the infant gut microbiome with early childhood neurodevelopmental outcomes an ancillary study to the VDAART randomized clinical trial. *JAMA Netw. Open* 2:905. 10.1001/jamanetworkopen.2019.0905 30901046 PMC6583279

[B86] Squires J. Twombly E. Bricker D. Potter L. (2009). *Excerpted from: ASQ-3 user’s guide.* Available online at: www.agesandstages.com (accessed March 12, 2022)

[B87] Stewart C. J. Ajami N. J. O’brien J. L. Hutchinson D. S. Smith D. P. Wong M. C. (2018). Temporal development of the gut microbiome in early childhood from the TEDDY study. *Nature* 2018:617. 10.1038/s41586-018-0617-x 30356187 PMC6415775

[B88] Straub L. Bateman B. T. Hernandez-Diaz S. York C. Lester B. Wisner K. L. (2022). Neurodevelopmental disorders among publicly or privately insured children in the united states. *JAMA Psychiatry* 79 232–242. 10.1001/JAMAPSYCHIATRY.2021.3815 34985527 PMC8733868

[B89] Streit F. Prandovszky E. Send T. Zillich L. Frank J. Sabunciyan S. (2021). Microbiome profiles are associated with cognitive functioning in 45-month-old children. *Brain. Behav. Immun.* 2021:1061. 10.1016/J.BBI.2021.08.001 34371134

[B90] Syme C. Pelletier S. Shin J. Abrahamowicz M. Leonard G. Perron M. (2019). Visceral fat-related systemic inflammation and the adolescent brain: a mediating role of circulating glycerophosphocholines. *Int. J. Obes.* 43 1223–1230. 10.1038/s41366-018-0202-2 30206338

[B91] Tamana S. K. Tun H. M. Konya T. Chari R. S. Field C. J. Guttman D. S. (2021). *Bacteroides*-dominant gut microbiome of late infancy is associated with enhanced neurodevelopment. *Gut Microbes* 13 1–17. 10.1080/19490976.2021.1930875 34132157 PMC8210878

[B92] Tamburini S. Shen N. Wu H. C. Clemente J. C. (2016). The microbiome in early life: implications for health outcomes. *Nat. Med.* 227 713–722. 10.1038/nm.4142 27387886

[B93] Tangney J. P. Baumeister R. F. Boone A. L. (2004). High self-control predicts good adjustment, less pathology, better grades, and interpersonal success. *J. Pers.* 72 271–324. 10.1111/J.0022-3506.2004.00263.X 15016066

[B94] Tommaso N. D. Gasbarrini A. Ponziani F. R. (2021). Intestinal barrier in human health and disease. *Public Health* 18:12836. 10.3390/ijerph182312836 34886561 PMC8657205

[B95] Tottenham N. Hare T. A. Quinn B. T. Mccarry T. W. Nurse M. Gilhooly T. (2010). Prolonged institutional rearing is associated with atypically large amygdala volume and difficulties in emotion regulation. *Dev. Sci.* 2010:852. 10.1111/j.1467-7687.2009.00852.x 20121862 PMC2817950

[B96] Turnbaugh P. J. Ley R. E. Hamady M. Fraser-Liggett C. M. Knight R. Gordon J. I. (2007). The human microbiome project. *Nature* 2007:6244. 10.1038/nature06244 17943116 PMC3709439

[B97] U.S. Department of Agriculture and U.S. Department of Health and Human Services (2020). *Dietary Guidelines for Americans, 2020–2025*, 9th Edn. Washington, DC: US Government Publishing Office. Available online at: https://www.dietaryguidelines.gov/

[B98] Valles-Colomer M. Falony G. Darzi Y. Tigchelaar E. F. Wang J. Tito R. Y. (2019). The neuroactive potential of the human gut microbiota in quality of life and depression. *Nat. Microbiol.* 44 623–632. 10.1038/s41564-018-0337-x 30718848

[B99] Vangay P. Ward T. Gerber J. S. Knights D. (2015). Antibiotics, pediatric dysbiosis, and disease. *Cell Host Microbe* 17 553–564. 10.1016/J.CHOM.2015.04.006 25974298 PMC5555213

[B100] Wang Y. Chen X. Yu Y. Liu Y. Zhang Q. Bai J. (2020). Association between gut microbiota and infant’s temperament in the first year of life in a chinese birth cohort. *Microorganisms* 8:753. 10.3390/microorganisms8050753 32429579 PMC7285300

[B101] Wu G. Zhang C. Wu H. Wang R. Shen J. Wang L. (2017). Genomic microdiversity of Bifidobacterium pseudocatenulatum underlying differential strain-level responses to dietary carbohydrate intervention. *mBio* 8:16. 10.1128/MBIO.02348-16 28196965 PMC5312088

[B102] Wu W. Zhao A. Liu B. Ye W. H. Su H. W. Li J. (2021). Neurodevelopmental outcomes and gut bifidobacteria in term infants fed an infant formula containing high Sn-2 palmitate: a cluster randomized clinical trial. *Nutrients* 13 1–14. 10.3390/NU13020693 33671493 PMC7926808

[B103] Zhang W. Sun Z. Zhang Q. Sun Z. Su Y. Song J. (2021). Preliminary evidence for an influence of exposure to polycyclic aromatic hydrocarbons on the composition of the gut microbiota and neurodevelopment in three-year-old healthy children. *BMC Pediatr.* 21:2539. 10.1186/s12887-021-02539-w 33596845 PMC7888120

[B104] Zhu J. Wang C. Qian Y. Cai H. Zhang S. Zhang C. (2022). Multimodal neuroimaging fusion biomarkers mediate the association between gut microbiota and cognition. *Prog. Neuropsychopharmacol. Biol. Psychiatry* 113 110468–110468. 10.1016/j.pnpbp.2021.110468 34736997

