# Supplementary material for: A systematic review on the impact of gastrointestinal microbiota composition and function on cognition in healthy infants and children
Source: Front Neurosci. 2023 Jun 14;17:1171970. doi: 10.3389/fnins.2023.1171970 (PMC10306408; doi:10.3389/fnins.2023.1171970)
Supplement: Supplementary file 1 [file Data_Sheet_1.docx]

Supplementary Material

**A Systematic Review on the Impact of Microbiota Composition and Function on Cognition in Healthy Infants and Children**

**Arden L. McMath^1^, Miriam Aguilar-Lopez^2,3^, Corinne N. Cannavale^4^, Naiman A. Khan^1,4,5,6^, Sharon M. Donovan^1,7*^**

***Correspondence:**

Sharon M. Donovan, PhD, RD

339 Bevier Hall

905 S. Goodwin Avenue,

Urbana, IL 61801

Email: [sdonovan@illinois.edu](mailto:sdonovan@illinois.edu)

Phone: 217-333-2289

**Supplemental Table 1.** Study characteristics.

| **Author& Year** | **Country** | **Characteristics of the study population** | ***N* (*N* females)** | **Age at gut microbiome sample** | **Age at cognitive assessment** | **Cognitive assessment** | **Outcomes** |
| --- | --- | --- | --- | --- | --- | --- | --- |
| **Observational Studies** | | | | | | | |
| Aatsinki 2019 | Finland | Infants from the FinnBrain Birth Cohort Study; 4% of infants were <37 weeks GA. | 301 (142) | 2.5-mo | 6-mo | Infant Behavior Questionnaire- Revised Short Form | Parent-reported infant behavior of negative emotionality, surgency/extraversion, and regulation/orienting. |
| Aatsinki 2020 | Finland | Infants from the “Focus cohort”^a^ of the FinnBrain Birth Cohort Study. | 122 (57) | 2.5-mo | 8-mo | Attention to emotional faces | Comparison of infant attention to neutral versus emotional facial expressions via eye-tracking. |
| Acuna 2021 | Spain | Met the threshold for typical neurodevelopment (per the BSID-III) from the PREOBE observational study cohort. | 71 (26) | 18-mo | 18-mo | Bayley Scales of Infant and Toddler Development, Third Edition | Experimenter scores child on various tasks that measures cognitive, language and motor skills. |
| Callaghan 2019 | USA | Secondary analysis for a study exploring the effects of early life caregiving adversity on fear reactivity. Considered a "proof-of-concept" study;. 8 children were considered to be exposed to early adverse caregiving experience. 8 were controls, raised with their biological families. | 16 (12) | 5-11-yo | 5-11-yo | functional reactivity to fear faces | fMRI captured during exposure to neutral and emotional faces. |
| Carlson 2018 | United States | Healthy infants from two prospective longitudinal studies of early brain development at University of North Carolina. | 1-y= 89 (40)  (46 with sMRI)  2-y= 69  (27 with sMRI) | 1-yo | 1-yo  2-yo | 1. Mullen scales of early development 2. Brain structures via sMRI | 1. Motor skills, visual reception, expressive language, receptive language and early learning composite (similar to IQ) assessed by a trained experimenter. 2. Total gray matter, total white matter, total cerebrospinal fluid, intracranial volume, lateral ventricle volume and 90-regions of gray matter volumes. |
| Carlson 2021 | United States | All vaginally delivered, >37 weeks gestational age and exclusively breastfed until 1-mo; mothers did not use antibiotics 2 weeks before delivery and infants did not use antibiotics before 1-mo. | 1-y= 34 (11)  (31 with sMRI)  2-y= 33  (23 with sMRI) | 1-mo  1-yo | 1-mo  1-yo | 1. Non-social fear 2. Social fear 3. Infant Behavior Questionnaire- Revised, Fear subscale only 4. Brain structures related to fear | 1. Masks portion of the locomotor Laboratory Temperament Assessment; assessed child reactions to various masks worn by a research assistant. 2. “The Strange Situation” paradigm; assessed child reactions to stranger entering and exiting the room with the infant. 3. Parent-reported infant social and Non-social fear behaviors. 4. sMRI of the prefrontal cortex, amygdala and hippocampus. |
| Christian 2014 | United States | Recruited from general community of Columbus, Ohio. | 77 (36) | 18-27-mo | 18-27-mo | Early Childhood Behavior Questionnaire | Parent-reported negative affectivity, surgency/extraversion and effortful control. |
| Flannery 2020 | United States | Children were already in a larger study conducted in the Stress Neurobiology and Prevention laboratory. | 40 (23) | 5-7-yo | 5-7-yo | 1. Child behavior checklist 2. Child behavior questionnaire | 1. Parent-reported internalizing and externalizing behavioral problems. 2. Parent-reported total behavioral dysfunction |
| Fox 2021 | United States | Infants involved in a larger prospective, longitudinal cohort study (Pregnancy Experiences and Infant Development Study). | 67^a^ (32) | 1-3 wks  2, 6 and 12-mo | 12-mo | Infant Behavior Questionnaire- Revised | Parent-reported infant behavior of negative emotionality, surgency/extraversion, and regulation/orienting. |
| Gao 2019 | United States | Healthy infants from two prospective longitudinal studies of early brain development at University of North Carolina. | 39 (23) | 1-yo | 1-yo (MRI only)  2-yo (Mullen only) | 1. Mullen scales of early development 2. Functional connectivity of the amygdala | 1. Motor skills, visual reception, expressive language, receptive language and early learning composite (similar to IQ) assessed by a trained experimenter. 2. rsfMRI |
| Guzzardi 2022 | Italy | From the Italian Pisa birth cohort. | 90 (42) | First-pass meconium samples (mostly within 1^st^ 5-6 hours)  (n = 79)  3-mo (40)  6 (47)  12 (37)  36 (21) | 6-mo (n= 24)  12 (26)  18 (26)  24 (23)  36 (27)  60 (56) | Griffiths Mental Development Scales (6- 24-mo) and the Extended revised version (36-60-mo) | Locomotor skills, personal and social skills, hearing and language skills, eye and hand coordination, performance skills and practical reasoning skills were assessed by a trained psychologist. |
| Kelsey 2021 | United States | Term, newborns were recruited from a local academic medical center in Charlottesville, Virginia. | 63 (25) | 9-56 days | - 1. ays | 1. Infant Behavior Questionnaire- Revised Short Form 2. Functional connectivity of the prefrontal and parietal cortex | 1. Parent-reported infant behavior of negative emotionality, surgency/extraversion, and regulation/orienting. 2. fNIRS |
| Loughman 2020 | Australia | Infants from the Barwon Infant Study. Those with a fecal sample at 1, 6 or 12 mo were included. Participants had to be >32 weeks gestational age. | 182 at 1-mo  190 at 6-mo  201(95) at 12-mo | 1, 6, 12-mo | 2-yo | Child behavior checklist | Parent-reported internalizing and externalizing behavioral problems. |
| Michels 2019 | Belgium | Dutch-speaking Belgian children part of a larger longitudinal study, ChiBS; No participants had oral anti-inflammatory drugs or SSRIs 3 mo before the fecal sample. | 64 (NR) | 8-11-yo | 8-11-yo | 1. Strengths and Difficulties Questionnaire 2. Positive and negative emotions, Internally validated questionnaire | 1. Parent-reported emotional problems over the past 6-mo. 2. Participant-reported feelings the majority of the time; includes happiness, sadness, anxiety and anger. |
| Rothenberg 2021 | China | Infants from the Maternal and Child Health Hospital in Daxin County, Guiangxi province, China (predominantly rural). | 46 (18) | 36-mo | 36-mo | Bayley Scales of Infant Development, 2nd edition | Experimenter scores child on various tasks that measures mental and psychomotor development. |
| Sordillo 2019 | United States | Children from a larger 2-arm, double-blind, placebo controlled, randomized clinical trial. Women were recruited from Boston Medical Center, Washington University at St Louis and Kaiser Permanente Southern California Region (San Diego). | 309 (139) | 3-6 mo | 3.0-yo | Ages and Stages Questionnaire | Parent-reported motor skills, problem-solving ability, communication skills, personal and social skills. |
| Streit 2021 | Germany& United States | From an ongoing longitudinal study on pre, peri, and postnatal stress and offspring development and health; children had to be >30 gestational age. | 323 (172) | 45-mo | 45-mo | Wechsler Preschool and Primary Scale of Intelligence - Third Edition | Full-scale, verbal and performance IQ and language skills assessed by a trained experimenter. |
| Tamana 2021 | Canada | Infants from the Edmonton site of the CHILD Cohort Study Recruitment took place in Vancouver, Edmonton, Winnipeg and Toronto; infants had a gestational age of >35 weeks. | 414 at 4-mo  405 (198) at 12-mo | 4-mo  12-mo | 12-mo  24-mo | Bayley Scale of Infant Development Third Edition | Experimenter scores child on various tasks that measures cognitive, language and motor skills. |
| Wang 2020 | China | Healthy mothers and infants recruited during late pregnancy from obstetric outpatient clinics in a Grade III A Hospital of Wuhan University (Wuhan, Hubei Province). | 51 (31) | 12-mo | 12-mo | Infants Behavior Questionnaire-Revised-Chinese version | Parent-reported infant behavior of negative emotionality, surgency/extraversion, and regulation/orienting. |
| Zhang 2021 | China | Naturally delivered, breastfed, infants from a prospective birth cohort study. Infants had to have had polycyclic aromatic hydrocarbon exposure and had no antibiotic exposure in the last 30 days. | 38 (18) | 3-yo | 3-yo | 1. Child behavior checklist 2. Gessell Development Inventory | 1. Parent-reported internalizing and externalizing behavioral problems. 2. Adaptive behavior, motor skills, language skills, personal and social skills development assessed by a clinician. |
| **Randomized Controlled Trials** | | | | | | | |
| Sobko 2020 | China  (Hong Kong) | Children were recruited for a 10-week Play & Grow, outdoor play intervention at the University of Hong Kong.  30 children were randomized to an intervention group and 24 to a control group. Children did not use antibiotics in 2-mo prior to the start of the study. | 27 (14) intervention group  18 (8) control group | 10-week intervention | 36-mo | Perceived Stress Scale for Children | Joint reporting by parents and children of child stress related to home and school, behavior and health. |
| Wu 2021 | China | Breastfed and formula-fed infants were recruited from four obstetric units. When mothers unequivocally decided not to breastfeed, only then were they approached for inclusion in formula-fed groups. Formula-fed infants were randomized to receive a formula containing sn-2 palmitate enriched formula or a standard control formula. Infants on antibiotics were excluded from analyses and had to be >37 weeks gestational age. | 57 (21) Breast-fed infants  59 (30) control formula  58 (25) sn-2 formula | 7-14 days old  16 & 24-weeks old | 16 & 24-wks | 1. Ages and Stages Questionnaire | Parent-reported motor skills, problem-solving ability, communication skills, personal and social skills. |

^a^Total number of participants included; not all infants had samples at each timepoint: 23 at 1-3 wks; 25 at 2-mo; 16 at 6-mo; 27 at 12-mo.

Abbreviations: fMRI, functional magnetic resonance imaging; fNIRS, functional near-infrared spectroscopy; sMRI, structural magnetic resonance imaging; mo, months old; SSRI, selective serotonin reuptake inhibitor; yo, years old; IQ, intelligence quotient; NR, not reported

**Supplemental Table 2.** Microbiome characteristics associated with infant and child behavior and temperament.

| **Author & Year**  **(*N/N* female)** | **Alpha diversity** | **Beta diversity** | **Taxonomy** |
| --- | --- | --- | --- |
| **Effortful Control** | | | |
| Christian 2015^b^  77 (36) | - In ~24-mo females only, higher Shannon Diversity associated with lower effortful control - Phylogenetic Diversity not associated | - Weighted and Unweighted UniFrac PCoA clusters at ~24-mo not associated | - No significant relationships with effortful control or its subscales for males or females |
| Flannery 2020  40 (23) | Not determined (ND) | - Euclidean distances not associated with behavior dysfunction in children 5-7-yo | - Inhibitory control subscale positively associated with *Bacteroides fragilis*, and negatively with *Eubacterium siraeum, Bifidobacterium adolescentus*, and *Eubacterium rectale*. |
| **Negative Affect** | | | |
| Christian 2015^b^  77 (36) | - Shannon Diversity not associated with Negative Affect or its subscales at ~24-mo | - In females only, the fear subscale associated with Unweighted Unifrac, but not Weighted UniFrac at ~24-mo | - In females, fear positively associated with an undefined genus in the family *Rikenellaceae* at ~24-mo |
| Flannery 2020  40 (23) | ND | - Euclidean distances not associated with behavior dysfunction in children 5-7-yo | - Fear positively associated with *Parabacteroides distasonis*, *Bilophila* unclassified and *Roseburia intestinalis* - Sadness negatively associated with *Bacteroides fragilis*. - Anger/frustration positively associated with *Roseburia hominis* |
| Fox 2021  67 (32) | - 1-3 week, 2, 6 or 12-mo Chao1 or Shannon Diversity not associated with 12-mo temperament | - 12-mo PCoA clusters derived from Aitchison distances were associated with the sadness subscale at 12-mo, but not negative affect as a whole. - 1-3 week, 2 and 6-mo beta diversity not associated with negative affectivity or its subscales at 12-mo | - *Megamonas, Acidaminococcus* and *Ruminococcus-1* positively associated with negative affectivity at 12-mo - *Lactobacillus* genus negatively associated with negative affectivity at 12-mo |
| Wang 2020  51 (31) | ND | ND | - Analyses adjusting for delivery mode, feeding type, and probiotic consumption yielded no relationships between negative affect and relative abundances at 12-mo |
| Carlson 2021  1-y= 34 (11)  2-y= 33 (NR) | - Alpha diversity clusters at 1-mo and 1-yo not associated with parent-reported fear subscale at 1-yo - Higher alpha diversity at 1-mo associated with lower non-social fear behavior at 1-yo - Social fear behavior not associated with measures of alpha diversity | - 1-mo or 1-yo weighted and unweighted UniFrac not associated with 1-yo fear - 1-yo weighted UniFrac clusters characterized by higher abundances of *Veillonella, Dialister*, unnamed genus of *Clostridiales, Bifidobacterium* and *Lactobacillus*, but lower abundances of *Bacteroides* had higher non-social fear behavior | ND |
| Aatsinki 2019  301 (142) | - After adjusting for sex, mode of delivery, gestational age, breastfeeding and antibiotic usage, 2.5-mo Shannon diversity negatively associated with 6-mo negative emotionality and fear reactivity - Chao1 at 2.5-mo not associated with 6-mo temperament | - In analyses adjusted for sex and mode of delivery, Bray-Curtis distance-based Partitioning Around Medoids clusters at 2.5-mo were not associated with 6-mo negative affect - In partially adjusted analyses exploring interaction terms of cluster membership and sex, there was a significant sex difference in the association between 2.5-mo *Bifidobacterium/Enterobacteriaceae*- and *Bacteroides*-cluster membership for fear reactivity at 6-mo | - After adjusting for sex, mode of delivery, gestational age, breastfeeding and antibiotic usage, 2.5-mo genera not associated with 6-mo temperament - Several *Clostridiaceae* OTUs negatively associated with negative emotionality and fear reactivity - Females had negative associations of *Veillonella parvula* and *Veillonella dispar* OTUs with fear reactivity - One positive association with fear reactivity and another *Veillonella* OTU of unknown species also identified |
| Kelsey 2021  63 (25) | - Chao1 and Shannon Diversity not associated with temperament during early infancy | ND | - Above the median for *Bifidobacterium* (*B. pseudocatenulatum*) had significantly higher negative emotionality during early infancy - After adjusting for age, family income, breastfeeding, gestational age, head circumference and birthweight, the genus *Monilinia* and species*, Xenorhabdus cabanillasii, Rhodospirilum rubrum, Corynebacterium otitidis, Aquimonas voraii* positively associated - Negative associations identified for *Pseudomononas benzenivorans, Cytophaga aurantiaca, Rheihheimera baltica, Rhodoblastus acidophilus,Paracoccus saliphilus*^c^ |
| **Orienting/Regulation** | | | |
| Fox 2021  67 (32) | - 1-3 week, 2, 6 or 12-mo Chao1 and Shannon Diversity not associated with temperament at 12-mo | - 1-3 week, 2, 6 or 12-mo beta diversity not associated with 12-mo temperament trait or subscales | ND |
| Wang 2020  51 (31) | ND | ND | - After adjusting for delivery mode, feeding type and prebiotic consumption, *Bifidobacterium* positively related to soothability subscale at 12-mo - Cuddliness subscale also negatively associated with abundance of *Hungatella* after adjustment |
| Kelsey 2021  63 (25) | - Chao1 and Shannon Diversity not associated with temperament during early infancy | ND | - Above the median for *Bifidobacterium* (*B. pseudocatenulatum* and *B. catenulatum*) associated with higher regulation/orienting during early infancy - After adjusting for birthweight, income, breastfeeding, gestational age and head circumference, genus Monilinia and species *Rhodospirilum rubrum*, ,*Corynebacterium otitidis,* and *Aquimonas voraii.* positively associated with orienting/regulation - Negative associations observed for *Cytophaga aurantiaca, Rheihheimera baltica, Rhodoblastus acidophilus, Xenorhabdus cabanillasii, Pseudomononas benzenivorans*, and *Paracoccus saliphilus*^c^ |
| Aatsinki 2019  301 (142) | - In analyses adjusted for sex, mode of delivery, gestational age, breastfeeding and antibiotic usage, 2.5-mo alpha diversity not associated with 6-mo temperament | - In models adjusted for age, sex and mode of delivery, the *Bacteroides* cluster negatively associated with regulation, and its subscale, duration of orienting, upon comparison to *Bifidobacterium/ Enterobacteriaeae* cluster - *Veillonella dispar* cluster also negatively associated with regulation and cuddliness subscale in comparison to *Bifidobacterium/ Enterobacteriaeae* cluster | - After adjustment for age, sex, mode of delivery gestational age, breastfeeding and antibiotic usage, *Erwinia* positively associated with regulation - *Veillonella* OTUs both positively and negatively associated with regulation - *Bifidobacterium* OTU positively associated with regulation - For males, regulation negatively associated with OTUs of *Veillonella dispar* and *parvula*) and positively with *Bifidobacterium* and *Clostridiaceae*. |
| **Surgency/**  **Extraversion** |  |  |  |
| Aatsinki 2019  301 (142) | - For analyses adjusted for sex, mode of delivery, gestational age, breastfeeding and antibiotic usage, 2.5-mo alpha diversity not associated with 6-mo temperament | - In analyses adjusted for sex and mode of delivery, 2.5-mo cluster membership not associated with 6-mo surgency or its subscales | - For analyses adjusted for sex, mode of delivery, gestational age, breastfeeding and antibiotic usage, 2.5-mo *Streptococcus* positively associated with 6-mo surgency - Two *Veillonella dispar* OTUs negatively associated with surgency - One *Bifidobacterium* OTU positively associated with surgency - Males demonstrated positive associations with 6-mo surgency and several 2.5-mo *Bifidobacterium* OTUs and one *Lactobacillus zeae*.OTU |
| Kelsey 2021  63 (25) | - Chao1 and Shannon Diversity not associated with temperament in early infancy | ND | - Above and below median comparison not associated during early infancy - After adjusting for birthweight, income, breastfeeding, gestational age and head circumference, genus *Monilinia* and species *Pseudomononas benzenivorans, Rhodospirilum rubrum, Stagonosporopsis tanaceti, Cytophaga aurantiaca,Corynebacterium otitidis,* and *Aquimonas voraii* positively associated - Negative associations observed for *Rheihheimera baltica, Rhodoblastus acidophilus, Balamuthia mandrillaris, Xenorhabdus cabanillasii*, and *Paracoccus saliphilus*^c^ |
| Fox 2021  67 (32) | - 1-3 week, 2, 6 or 12-mo Chao1 and Shannon Diversity not associated with 12-mo temperament | - PCoA clusters of Aitchison distance at 1-3 weeks associated with surgency at 12-mo, as well as its subscales approach, high-intensity pleasure and smiling/laughter - 2, 6 and 12-mo beta diversity not associated with surgency or its subscales at 12-mo | - At 1-3 weeks, genus *Bifidobacterium*, an unclassified *Lachnospiraceae*, and *Collinsella* positively associated with the surgency at 12-mo - At 1-3 weeks, genus *Klebsiella* negatively associated with surgency at 12-mo |
| Wang 2020  51 (31) | ND | ND | - Surgency and vocal reactivity subscale positively associated with *Faecalibacterium* and *Akkermansia* at 12-mo - Analyses adjusted for delivery mode, feeding type, and probiotic consumption yielded no associations |
| Christian 2015^b^  77 (36) | - In males, higher surgency associated with greater phylogenetic diversity, but not Shannon Diversity at ~24-mo - In females, higher surgency associated with higher phylogenetic diversity, but not Shannon Diversity at ~24-mo | - In males, surgency associated with unweighted Unifrac but not weighted UniFrac at ~24-mo - Subscales sociability, high-intensity pleasure and activity level associated with unweighted UniFrac - Only high-intensity pleasure subscale associated with weighted Unifrac. | - In males, high-intensity pleasure and activity level subscales positively associated with the genus *Dialister* and an undefined genus in the family *Rikenellaceae* at ~24-mo - Surgency not associated with taxa in females |
| Flannery 2020  40 (23) | ND | - Not associated with behavior dysfunction in children 5-7-yo | - Impulsivity negatively associated with *Bacteroides fragilis*, and positively associated with *Eubacterium siraeum* and *Bacteroides xylanisolvens* |
| **Internalizing behavior problems** | | | |
| Flannery 2020  40 (23) | ND | - Not associated with behavior dysfunction in children 5-7-yo | - Internalizing behavior negatively associated with *Bacteroides thetaiotaomicron* - Anxious depressed subscale negatively associated with *Veillonell*a unclassified and *Akkermansia muciniphila,* while it was positively associated with *Eubacterium siraeum, Bifidobacterium* *adolescentis*, and *Coprococcus comes* - Anxiety problems negatively associated with *Bacteroides thetaiotaomicron* and positively with *Bilophila unclassified* |
| Loughman 2020  182 (NR) at 1-mo  190 (NR) at 6-mo  201 (95) at 12-mo | ND | ND | - *Prevotella* abundance at 12-mo negatively associated with internalizing behavior problems at 2-yo - *Lachnospiraceae* family not associated with internalizing behaviors |
| Zhang 2021  38 (18) | ND | ND | - At 3-yo, *Bacteroides vulgatus, Bacteroides intestinalis, Bacteroides uniformis*, and *Bacteroides caccae* positively associated with most internalizing behavior problems - *Prevotellacceae,* Verrucomicrobia*, Lactobacillus* and *Anaerostipes* negatively associated with total internalizing behavior problems^d^ |
| **Externalizing behavior problems** | | | |
| Flannery 2020  40 (23) | ND | - Not associated with behavior dysfunction in children 5-7-yo | - In 5-7-yo, externalizing behavior positively associated with *Eubacterium siraeum, Bifidobacterium adolescentis* and *Bilophila* unclassified, while negatively associated with *Bacteroides thetaiotamicron, Bacteroides fragilis, Streptococcus salivarius,* *Haemophilus para influenzae* and *Veillonella* unclassified - Aggressive behavior subscale negatively associated with *Bacteroides fragilis*, *Bacteroides uniformis, Streptococcus salivarius,* and *Veillonella* unclassified, while positively associated with *Eubacterium siraeum* and *Bifidobacterium adolescentis*. - Emotionally reactive subscale positively associated with *Eubacterium siraeum,* while negatively associated with *Veillonella* unclassified, *Bacteroides fragilis* and *Streptococcus salivarius.* |
| Loughman 2020  182 (NR) at 1-mo  190 (NR) at 6-mo  201 (95) at 12-mo | ND | ND | - *Prevotella* abundance at 12-mo negatively associated with externalizing behavior problems at 2-yo - *Lachnospiraceae* family not associated with externalizing behaviors |
| Zhang 2021  38 (18) | ND | ND | - At 3-yo, Actinobacteria, *Bacteroides vulgatus, Bacteroides intestinalis, Bacteroides uniformis*, and *Bacteroides caccae* positively associated with externalizing behavior problems - *Prevotellacceae,*Verrucomicrobia*, Lactobacillus,*and *Anserostipes* negatively associated with externalizing behavior problems^d^ |
| **Total behavioral problems** | | | |
| Flannery 2020  40 (23) | ND | - Not associated with behavior dysfunction in children 5-7-yo | - In 5-7-yo, total behavioral problems positively associated with *Eubacterium siraeum,* and negatively with *Veillonella* unclassified, *Bacteroides fragilis, Bacteroides thetaiotamicron*, and *streptococcus salibarius* |
| Loughman 2020  182 (NR) at 1-mo  190 (NR) at 6-mo  201 (95) at 12-mo | - 1 and 6-mo Shannon diversity not associated with 2-yo behavior outcomes | - 1 and 6-mo weighted and unweighted UniFrac distances not associated with 2-yo behavior outcomes - 12-mo unweighted UniFrac distances, but not weighted, significantly different between high and low behavioral problem groups | - 1-mo relative abundances not different for low high versus low behavioral problem groups - 12-mo *Prevotella* abundance negatively associated with elevated behavioral problems at 2-yo, even after adjustment for storage variables, posteriori-selected variables^a^, and 1, 6, and 12-mo temperament traits - 12-mo *Lachnospiraceae* abundance positively associated with elevated behavioral problems at 2-yo, although this was attenuated upon adjustment for covariates |
| Zhang 2021  38 (18) | ND | ND | - At 3-yo, Verrucomicrobia, Verrucomicobiae, Rhodospirillales, *Rhodospirillaceae*, *Akkermansia,* and *Sutterella* negatively associated with total behavioral problems - Bacteroidetes, Bacteroidia, *Clostridium colinum, Bacteroides intestinalis, Lactococcus garviieae, Bacteroides vulgatus, Clostridium lavalense, Roseburia inulinivorans, Clostridia bacterium* UC512F7 and *Clostridium perfringens* positively associated with total behavioral problems^d^ |
| **Emotional problems** | | | |
| Michels 2019  64 (NR) | - Observed species, Chao1 and Simpson diversity not associated with parent-reported emotional problems - Within preadolescents only, negative self-reported emotions associated with higher observed species - Chao1 and Simpson’s Diversity not associated with positive or negative self-reported emotions. | - Weighted and unweighted UniFrac distances not associated with parent-reported emotional problems - PCoA clusters for weighted and unweighted UniFrac distances different between low and high self-reported happiness subscale | - Negative emotions were positively associated with several *Veillonellaceae* and negatively with *Lachnospiraceae* (*Blautia*) - Happiness was positively associated with *Bacteroides* and *Prevotella*_UCG_001, but negatively associated with multiple Clostridiales, including multiple *Lachnospiraceae (Blautia, Roseburia, Lachnoclostridium*, NK4A136 and FCS020 groups), multiple Ruminoccocceae OTUs, *Akkermansia* and *Eubacterium* |
| **Perceived stress** | | | |
| Sobko 2020^e^  27 (14) intervention group  18 (8) control group | - Perceived stress and alpha diversity not associated, nor was there a time effect for alpha between intervention group and control group - Perceived stress negatively associated with Chao1 richness, Shannon Diversity and Simpson Diversity of Bacteroidetes - Outdoor activities intervention group had a significant decrease in overall stress scores compared to control, and those with significantly decreased stress scores in the intervention group exhibited significantly higher Chao1 richness compared to those that did not exhibit changes in stress scores | - Pre and post intervention community structure significantly different in both intervention and control groups - Anger frequency associated with community structure in the intervention but not control group - Bray distance at species level of children with the highest anger frequency significantly different from children with lower anger frequency - Overall and other components of perceived stress not associated | - *Roseburia* other and *Roseburia faecis* abundances significantly higher in those with higher anger scores |

^a^Posteriori-selected variables were gestational age, mode of birth, antibiotic use during labor, breastfeeding at four weeks, number of siblings, household pet ownership, child sex, age at time of questionnaire completion, maternal prenatal smoking, maternal perceived stress and depressive symptoms and household income.

^b^ No correction for multiple comparisons made. Instead limited analyses to genera that made up at least 1% of the total sample by relative abundance. Resulting comparisons were made for top 20 genera for males and top 18 for females.

^c^ Note, these results were not discussed by authors. Top 50 with lowest q-values reported by authors, but only top 12 reported here because these related to each domain of temperament.

^d^ Most exact p-values not reported; largest effect size results are derived from visual inspection of supplemental material Spearman correlation heatmaps.

^e^ Reported results focus on relationships directly assessed between perceived stress and gut microbiome. Results reporting changes in microbiome during the intervention are discussed in the text. There was no significant difference in control versus intervention groups by child age, gender, sleep, fruit and vegetable intakes or demographics.

Abbreviations: mo, mo old, ND, not determined; yo, years old, IQ, intelligence quotient; NR, not reported.

**Supplemental Table 3.** Microbiome characteristics associated with infant and child language, motor and higher order functions

| **Author & Year** | **Alpha diversity** | **Beta diversity** | **Taxonomy** |
| --- | --- | --- | --- |
| **Adaptive Behaviors** | | | |
| Zhang 2021  38 (18) | ND | ND | - 3-yo *Christensenellacaeae, Ruminococcaceae, Ruminococcus, Ruminococcus*_spp_N15MGS57 and *Clostridium lavalense* positively, while *Fusobacteriaceae, Anaerostipes caccae* and *Clostridium spiroforme* negatively related to adaptative behavior^a^ |
| **Cognitive Skills^b^** | | | |
| Tamana 2021  414 (NR) at 4-mo  405 (198) at 12-mo | - Not directly assessed; 12-mo lowest performing children (Proteobacteria-dominant cluster) had the lowest Faith’s Phylogenetic diversity, Chao1, Shannon and Simpson Index, while the highest performers (Firmicutes-dominant cluster) had the highest | - 4-mo and 12-mo clusters not associated with 1 or 2-yo cognitive skills - 12-mo Firmicutes-dominant, followed by Bacteroidetes-dominant clusters associated with better cognitive skills compared to the Proteobacteria-dominant cluster at 2-yo - Firmicutes-dominant cluster had highest change score in cognitive skills from 1 to 2-yo, followed by the Bacteroidetes-dominant cluster | - 12-mo *Bacteroides* including *B. fragilis*, *B. uniformis* and unclassified *Bacteroides* were positively associated, while *mucilaginosa* of the *Rothia* genus and an unclassified *Varibaculum* were negatively associated with cognitive skills at 2-yo |
| **Composite Cognitive Skills** | | | |
| Guzzardi 2022^c^  90 (42) | ND | - Higher composite cognitive development at 60-mo associated with first-pass meconium composition higher in *Bifidobacterium* - Other gut microbiota timepoints, 3, 6, 12 and 36-mo beta diversity not associated with composite cognitive development at 6, 12, 18, 24, 36 or 60-mo | ND |
| Rothenberg 2021^d^  46 (18) | - 3-yo Shannon Diversity, Faith’s Phylogenetic Diversity and Pielou’s measure of evenness were not associated with the 3-yo mental development scale | - 3-yo mental development positively associated with coabundance factor characterized by higher *Faecalibacterium, Clostridium* cluster XIVa, *Gemmiger, Phasolarctobacterium, Alstipes, Oscillibacter,* and *Sutterella*, and lower Blautia, *Anaerostipes, Clostridium* cluster XVIII, and *Streptococcus* | - *Faecalibacterium* and *Flavonifractor* positively correlated, while *Lachnospiraceae incertae sedis* negatively associated with mental development - After adjusting for multiple comparisons, associations were no longer present |
| Acuna 2021^e^  71 (26) | - 18-mo intra-sample, Faith's phylogenetic and Shannon's Diversity not significantly different for above and below median groups for composite cognition | - Weighted and unweighted UniFrac not significantly different between above and below median scores for composite cognition - Enterotype membership not associated with composite cognition | ND |
| Carlson 2018^f^  1-y= 89 (40)  2-y= 69 (NR) | - 2-yo early learning composite negatively associated with 1-yo Chao1, observed species, and Faith’s Phylogenetic diversity; inclusion of beta diversity as a covariate abrogated the relationship - 1-yo early learning composite not associated with 1-yo alpha diversity | - 1-yo community clusters characterized by high abundance of *Bacteroides* had the highest performance for early learning composite at 2-yo, while *Faecalibacterium*-dominant structures had the lowest performance, even after adjusting for Chao1, Faith’s Phylogenetic Diversity and Shannon Diversity as covariates; inclusion of Observed Species abrogated the relationship - 1-yo early learning composite not associated with 1-yo beta diversity - Change score in early learning from 1 to 2-yo not associated with beta diversity | ND |
| Streit 2021^g^  323 (172) | - 4-yo full-scale IQ negatively associated with Faith phylogenetic diversity index before, but not after, adjustment for multiple comparisons - Observed OTUs, Shannon Diversity, and Pileou’s Evenness were not associated | - | - 4-yo abundance of an unidentified genus within the family *Enterobacteriaceae* (most closely related to *Enterobacter asburiae, Enterbacter cloacae*, and *Kluyvera intermedia*) associated with lower full scale IQ |
| **Hearing and Language Skills** | | | |
| Guzzardi 2022  90 (42) | ND | - First-pass meconium, 3, 6, 12, 36-mo Weighted and Unweighted UniFrac diversity not associated with hearing and language skills at 6, 12, 18, 24, 36 or 60-mo | ND |
| **Language Skills** | | | |
| Acuna 2021  71 (26) | - Intra-sample, Faith's phylogenetic and Shannon's Diversity not significantly different for above and below median groups for language skills | - Weighted and unweighted UniFrac not significantly different between above and below median scores for language - Enterotype membership not associated with language | ND |
| Carlson 2018  1-y= 89 (40)  2-y= 69 (NR) | - At 2-yo, negative associations observed for expressive language with Chao1, Observed Species and Faith’s Diversity Index - Change in performance from 1 to 2-yo significantly different between alpha diversity measures for receptive language and expressive language, even after adjustment for beta diversity | - 1-yo clustering characterized by *Bacteroides* had the highest expressive and receptive language skills at 2-yo, while clusters characterized by *Faecalibacterium* had the lowest; inclusion of alpha diversity measures, except for Observed Species, did not abrogate this relationship - Clusters dominated by *Bacteroides* also had significantly larger change scores for both language scores from 1 to 2-yo, compared to the *Faecalibactterium* or *Rumimococcaceae* clusters - No associations for language scores at 1-yo | ND |
| Streit 2021  323 (172) | - 4-yo language skills negatively associated with Faith phylogenetic diversity index before, but not after, adjustment for multiple comparisons - Observed OTUs, Shannon Diversity, and Pileou’s Evenness not associated | ND | - 4-yo abundance of an unidentified genus within the family *Enterobacteriaceae* (most closely related to *Enterobacter asburiae, Enterbacter cloacae*, and *Kluyvera intermedia*) associated with lower language skills |
| Tamana 2021  414 (NR) at 4-mo  405 (198) at 12-mo | - Not directly assessed; 12-mo lowest performing children (Proteobacteria-dominant cluster) had the lowest Faith’s Phylogenetic diversity, Chao1, Shannon and Simpson Index, while the highest performers (Firmicutes-dominant cluster) had the highest | - 4- and 12-mo clusters not associated with 1 or 2-yo language skills - 12-mo Bacteroidetes-dominant, followed by Firmicutes-dominant clusters associated with better language skills compared to the Proteobacteria-dominant cluster at 2-yo - Bacteroidetes-dominant cluster also had highest change score in cognitive skills from 1 to 2-yo, followed by the Firmicutes-dominant cluster | - 12-mo *Bacteroides uniformis* and unclassified *Bacteroides* were positively associated, while *mucilaginosa* of the *Rothia* genus and unclassified Actinomyces negatively associated with language skills at 2-yo |
| Zhang 2021  38 (18) | ND | ND | - In 3-yo children, some of the strongest positive relationships existed for *Bifdobacteriaceae,, Bifidobacterium, Bifidobacterium longum, Bacteroides vulgatus,* and *Chryseobacterium hominis,* while some of the strongest negative relationship existed for *Verrucomicrobiaceae Tyzzerella,Lactobacillus delbrueckii* and *Bacteroides stercoris* with language skills^a^ |
| Sordillo 2019^h^  309 (139) | - 3-6-mo Shannon Diversity not associated with 3-yo communication skills | - Coabundance factor characterized by hjgh *Lachnospiraceae* genera and unclassified Clostridiales taxa and low *Bacteroides* associated with lower communication skills | - Communication negatively associated with Clostridiales, especially *Lachnospiraceae Ruminococcus* |
| Wu 2021  57 (21) Breast-fed infants  59 (30) control formula  58 (25) sn-2 formula | ND | ND | - Relative abundance of Bifidobacteria at 16-weeks-of-age not associated with communication skills |
| Streit 2021^i^  323 (172) | - 4-yo verbal IQ negatively associated with Faith phylogenetic diversity index before, but not after, adjustment for multiple comparisons - Observed OTUs, Shannon Diversity, and Pileou’s Evenness not associated | ND | - 4-yo abundance of an unidentified genus within the family *Enterobacteriaceae* (most closely related to *Enterobacter asburiae, Enterbacter cloacae*, and *Kluyvera intermedia*) and *Eubacterium* genus of the *Erysipelotrichaceae* family associated with lower verbal IQ |
| **Motor Skills** | | | |
| Wu 2021  57 (21) Breast-fed infants  59 (30) control formula  58 (25) sn-2 formula | ND | ND | - Relative abundance of Bifidobacteria at 16-weeks-of-age not associated with fine or gross motor skills. |
| Guzzardi 2022  90 (42) | ND | - First-pass meconium, 3, 6, 12, 36-mo Weighted and Unweighted UniFrac diversity not associated with locomotor skills at 6, 12, 18, 24, 36 or 60-mo | ND |
| Acuna 2021  71 (26) | - Intra-sample (richness), Faith's phylogenetic and Shannon's Diversity not significantly different for above and below median groups for fine or gross motor skills | - Weighted, but not unweighted, UniFrac clusters significantly different between above and below median scores for fine motor skills - Enterotype (based on genus level) characterized by higher Firmicutes had higher fine motor scores compared to those belonging to the enterotype characterized by *Bacteroides* - No associations observed for gross motor skills | - 18-mo children with fine motor skills above the median had higher abundances of *Bifidobacterium, Collinsella, Coprococcus, Enterococcus, Fusobacterium, Lactobacillus, Roseburia, Veillonella*, and an unassigned genus within *Veillonellaceae* - *Parabacteroides* more abundant in the children scoring below the median for fine motor skills - Of the 7 *Bifidobacterium* OTUs explored, an OTU with 100% sequence identity with *B. bifidum* ATCC 29521 tended to be enriched in children with scores above the median for fine motor skills |
| Carlson 2018  1-y= 89 (40)  2-y= 69 (NR) | - Chao1, observed species, Shannon index and Faith’s Phylogenetic Diversity not associated with 1 or 2-yo fine or gross motor skills | - 1 and 2-yo did not cluster differently based on their fine or gross motor skills - Change score for motor skills between 1 and 2-yo not significantly different between clusters | ND |
| Rothenberg 2021  46 (18) | - 3-yo Shannon Diversity, Faith’s Phylogenetic Diversity and Pielou’s measure of evenness not associated with the 3-yo motor development scale | - 3-yo motor skills positively associated with higher *Faecalibacterium, Clostridium* cluster XIVa, *Gemmiger, Phasolarctobacterium, Alstipes, Oscillibacter*, and *Sutterella*, and lower *Blautia, Anaerostipes, Clostridium* cluster XVIII, and *Streptococcus* | - *Faecalibacterium* and *Gemmiger* positively associated with motor development - After adjusting for multiple comparisons, no association between taxa and motor development |
| Sordillo 2019  309 (139) | - 3-6-mo Shannon Diversity not associated with 3-yo motor skills | - Coabundance factor characterized by high *Bacteroides* and low *Escherichia/Shigella* and *Bifidobacterium* associated with lower fine motor skills - No associations observed for gross motor skills | - Fine motor skills at 3-yo associated negatively with 3-6-mo *Enterobacteriaceae* abundance, especially *Klebsiella* |
| Tamana 2021  414 (NR) at 4-mo  405 (198) at 12-mo | - Not directly assessed; 12-mo lowest performing children (Proteobacteria-dominant cluster) had the lowest Faith’s Phylogenetic diversity, Chao1, Shannon and Simpson Index, while the highest performers (Firmicutes-dominant cluster) had the highest | - 4- and 12-mo clusters not associated with 1 or 2-yo motor skills - 12-mo Bacteroidetes-dominant, followed by Firmicutes-dominant clusters associated with better motor skills compared to the Proteobacteria-dominant cluster at 2-yo - 12-mo clusters not associated with motor skills | - 12-mo unclassified *Prevotella* and *Bacteroides fragilis* positively associated, while unclassified *Actinomyces* and unclassified *Methylophilaceae* negatively associated with motor skills at 2 yo |
| Zhang 2021  38 (18) | ND | ND | - 3-yo *Ruminococcaceae, Ruminicoccaceae_bacterium,,* *Desulfovibrionaceae, Hungatella, Bilophila* and *Veillonellaceae_bacterium* were among the strongest positive relationships, while *Clostridiaceae, Clostridium spiroforme, Paraclostridium, Paraclostridium bifermentan* were among the most negatively relationships with gross motor skills - *Ruminococcaceae, Coproccoccus eutactus, Faecalibacterium, Dialister, Chryseobacterium, Chryseobacterium hominis* and *Bifidobacteriaeceae* demonstrated some of the strongest positive, while *Prevotellaceae,* *Fusobacteriaceae, Fusobacterium,* uncultured *Clostridiales_bacterium,* and *Lactobacillus delbrueckii* showed the strongest negative relationship with fine motor skills^a^ |
| Guzzardi 2022^j^  90 (42) | ND | - First-pass meconium, 3, 6, 12, 36-mo Weighted and Unweighted UniFrac diversity not associated with hand-eye coordination skills at 6, 12, 18, 24, 36 or 60-mo | ND |
| **Performance Skills^k^** | | | |
| Streit 2021  323 (172) | - Observed Species, Shannon and Faith’s Diversity Indexes, and Pileou’s Evenness not associated with Performance IQ | ND | - 4-yo abundance of an unidentified genus within the family *Enterobacteriaceae* (most closely related to *Enterobacter asburiae, Enterbacter cloacae*, and *Kluyvera intermedia*) associated with lower performance IQ |
| Guzzardi 2022  90 (42) | ND | - First-pass meconium, 3, 6, 12, 36-mo Weighted and Unweighted UniFrac diversity not associated with performance skills at 6, 12, 18, 24, 36 or 60-mo | ND |
| **Personal and Social Skills** | | | |
| Sordillo 2019  309 (139) | - 3-6-mo Shannon Diversity not associated with 3-yo personal and social skills | - Coabundance factor characterized by high *Lachnospiraceae* genera and unclassified Clostridiales taxa and low *Bacteroides* associated with lower personal and social skills | - Personal and social skills negatively associated with taxa in the Clostridiales order |
| Zhang 2021  38 (18) | ND | ND | - *Parabacteroides, Collinsella* and *Campylobacteraceae* exhibited some of the strongest positive, while *Veillonellaceae, Paraclostridium, Paraclostrium bifermentans,, Lactococcus, Lactococcus garvieae,* and *Dialister* exhibited the strongest negative relationship with personal and social skills^a^ |
| Wu 2021  57 (21) Breast-fed infants  59 (30) control formula  58 (25) sn-2 formula | ND | ND | - Relative abundance of Bifidobacteria at 16-weeks-of-age not associated with personal and social skills |
| Guzzardi 2022  90 (42) | ND | - First-pass meconium, 3, 6, 12, 36-mo Weighted and Unweighted UniFrac diversity not associated with personal and social skills at 6, 12, 18, 24, 36 or 60-mo | ND |
| **Practical Reasoning Skills** | | | |
| Guzzardi 2022  90 (42) | ND | - Those with first-pass meconium weighted UniFrac characterized by higher *Bifidobacterium* and *Veillonella* abundances had better practical reasoning skills, showing a trend at 36-mo and reaching significance by 60-mo | ND |
| **Problem-solving Ability** | | | |
| Sordillo 2019  309 (139) | - Shannon Diversity at 3-6-mo not associated with 3-yo problem-solving | - Coabundance factors not associated with problem-solving abilities | ND |
| Wu 2021  57 (21) Breast-fed infants  59 (30) control formula  58 (25) sn-2 formula | ND | ND | - Relative abundance of Bifidobacteria at 16-weeks-of-age not associated with problem-solving ability |
| **Visual Reception** | | | |
| Carlson 2018  1-y= 89 (40)  2-y= 69 (NR) | - Visual reception negatively associated with Chao1, Observed Species and Shannon Index, but not Faith’s Phylogenetic Diversity | - 1 and 2-yo did not cluster differently based on their visual reception skills - Change score for visual reception skills between 1 and 2-yo not significantly different between clusters | ND |

^a^Most exact p-values not reported; largest effect size results are derived from visual inspection of supplemental material Spearman correlation heatmaps. Only family level relationships and below and those discussed in the text are reported in the table.

^b^From Bayley Scales of Infant and Toddler Development, Third Edition; includes measures of visual preference, attention, exploration, manipulation, and concept formation.

^c^From Griffiths Mental Development Scales; includes locomotor, personal and social, hearing and language, eye and hand coordination, performance and practical reasoning skills (the latter skill was only included for 36-mo and older).

^d^From Bayley Scales of Infant and Toddler Development, Second Edition; represents a composite of cognitive and language skills from Bayley Scales of Infant Development, Third Edition.

^e^From Bayley Scales of Infant and Toddler Development, Third Edition; composite score that includes cognitive, language, motor, social-emotional and adaptive behavior skills.

^f^A composite score from Mullen Scales of Early Development; includes subscales of language, fine motor and visual reception.

^g^From Wechsler Preschool and Primary Scale of Intelligence, Third Edition; represents composite of verbal, performance and language subscales.

^h^Language and communication skills.

^i^Verbal IQ.

^j^Eye-hand coordination skills.

^k^Measures fine motor manipulation skills and visual spatial orientation.

Abbreviations: mo, mo old; ND, not determined; yo, years old; IQ, intelligence quotient; NR, not reported.

**Supplemental Table 4.** Quality of the included studies by the NIH Quality Assessment Tool for Observational Cohort and Cross-sectional Studies.

| **Study** | Was the research question or objective in this paper clearly stated? | Was the study population clearly specified and defined? | Was the participation rate of eligible persons at least 50%? | Were all the subjects selected or recruited from the same or similar populations? | Was a sample size justification, power description, or variance and effect estimates provided? | For the analyses in this paper, were the exposure(s) of interest measured prior to the outcome(s) being measured? | Was the timeframe sufficient so that one could reasonably expect to see an association between exposure and outcome if it existed? | For exposures that can vary in amount or level, did the study examine different levels of the exposure? | Were the exposure measures (independent variables) clearly defined, valid, reliable, and implemented consistently across all study participants? | Was the exposure(s) assessed more than once over time? | Were the outcome measures (dependent variables) clearly defined, valid, reliable, and implemented? consistently across all study participants? | Were the outcome assessors blinded to the exposure status of participants? | Was loss to follow-up after baseline 20% or less? | Were key potential confounding variables measured and adjusted statistically for their impact on the relationship? between exposure(s) and outcome(s)? | Summary Quality |
| --- | --- | --- | --- | --- | --- | --- | --- | --- | --- | --- | --- | --- | --- | --- | --- |
| Aatsinki  2020 | 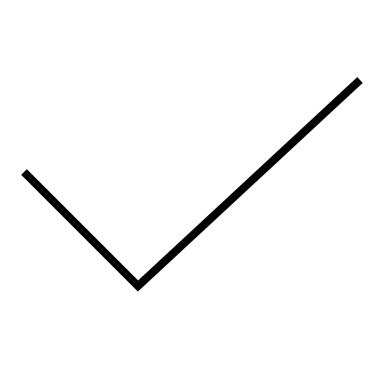 | 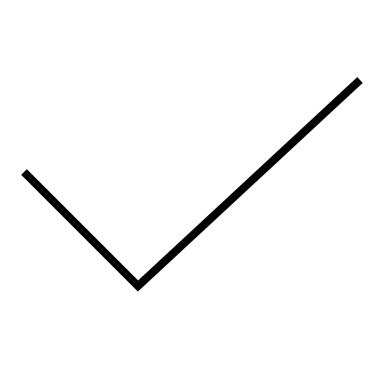 | 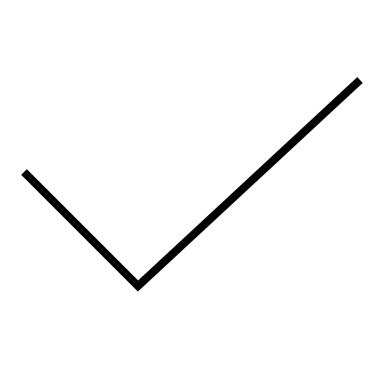 | 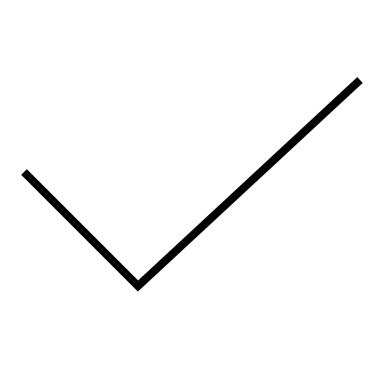 | 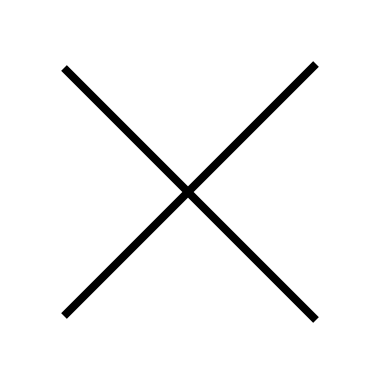 | NA | 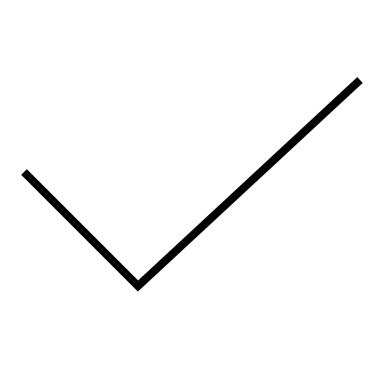 | 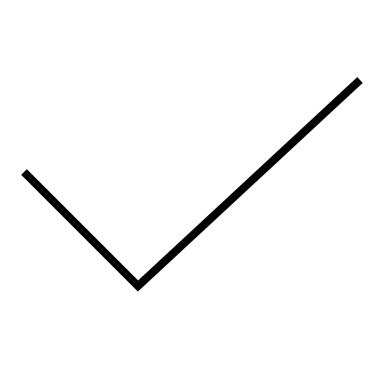 | 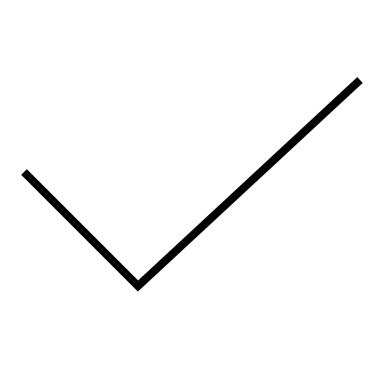 | 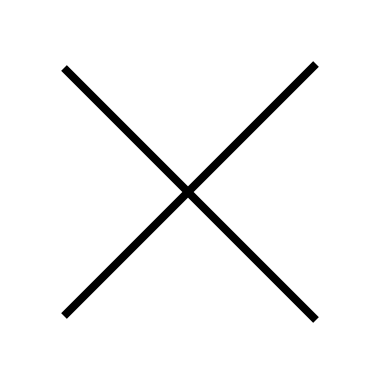 | 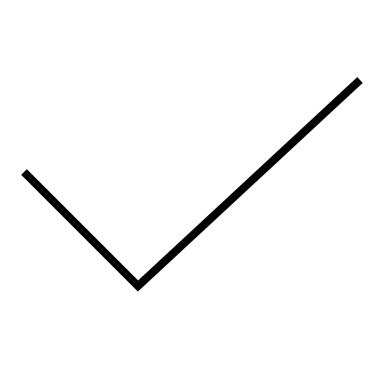 | NS | NS | 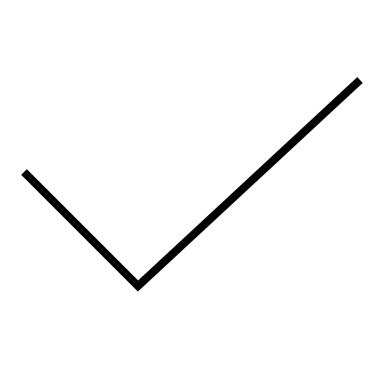 | F |
| Aatsinki 2019 | 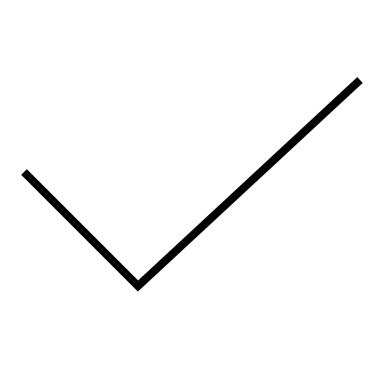 | 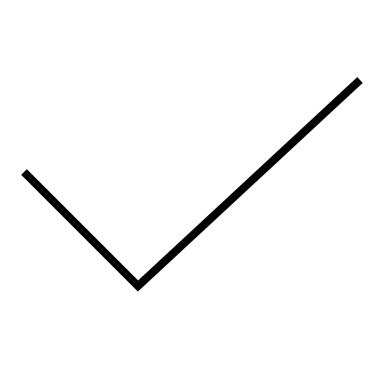 | 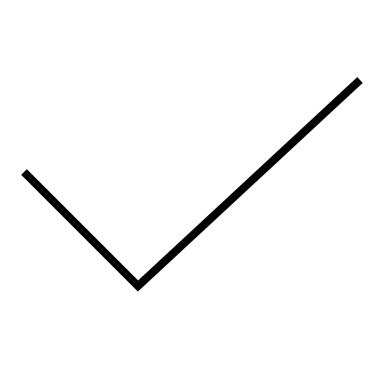 | 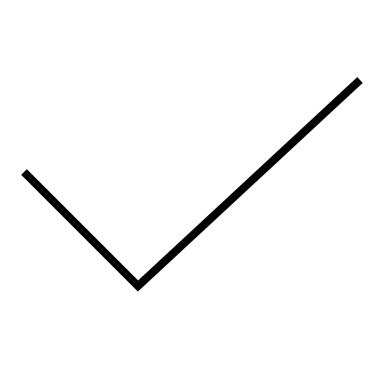 | 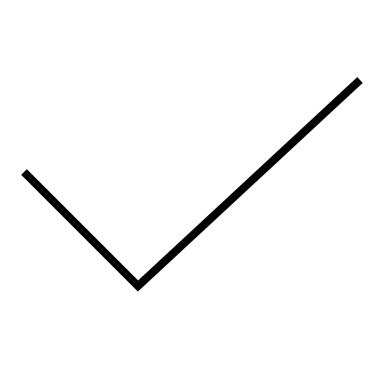 | NA | 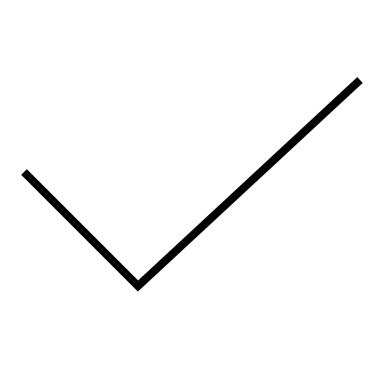 | 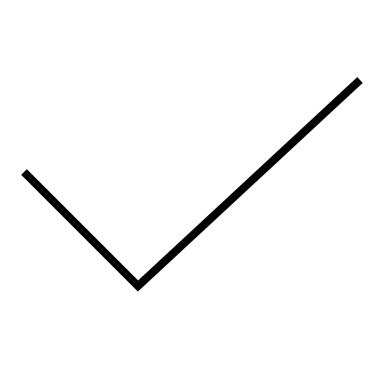 | 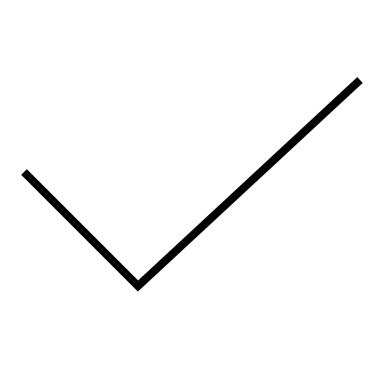 | 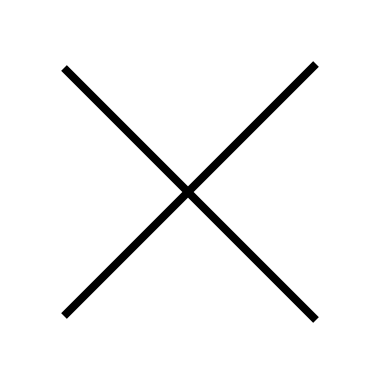 | 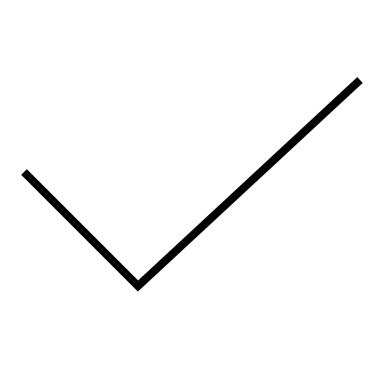 | NS | NS | 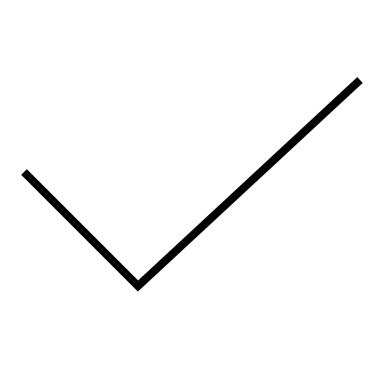 | F |
| Acuna | 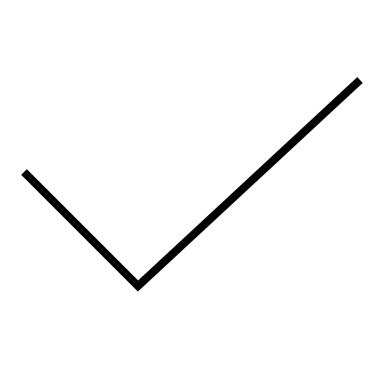 | 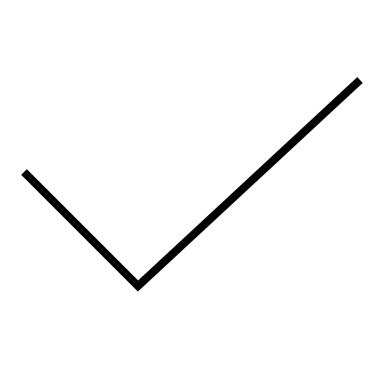 | 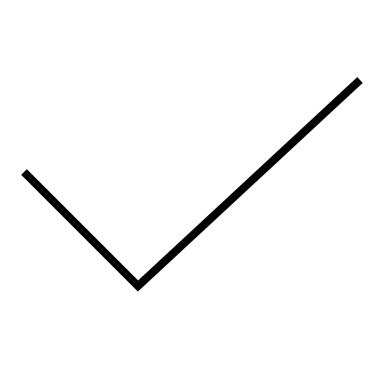 | 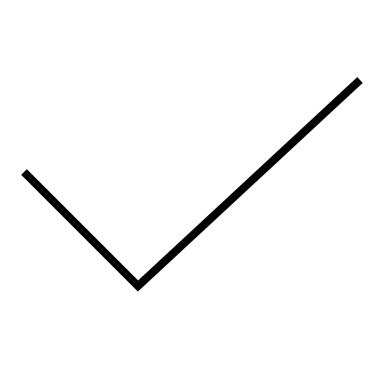 | 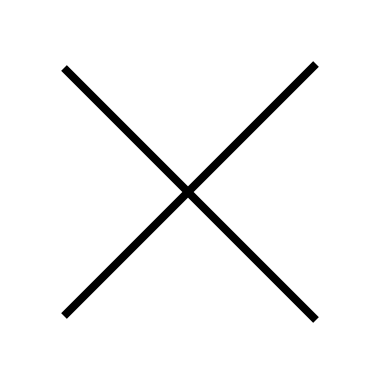 | 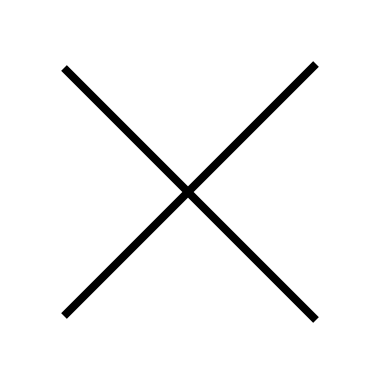 | 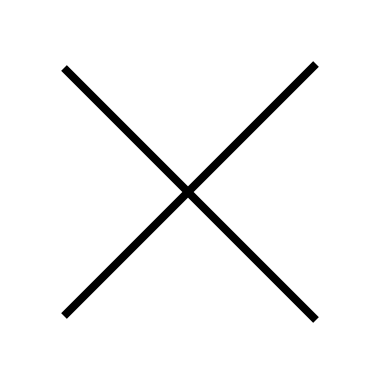 | 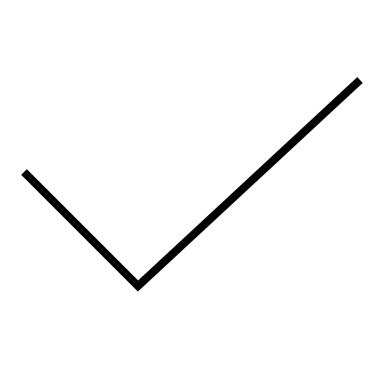 | 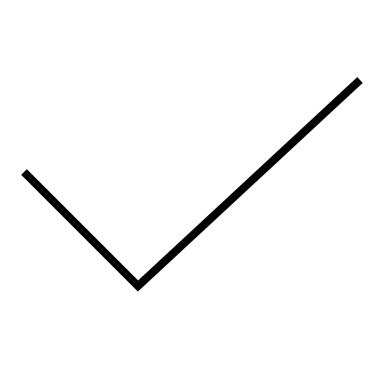 | 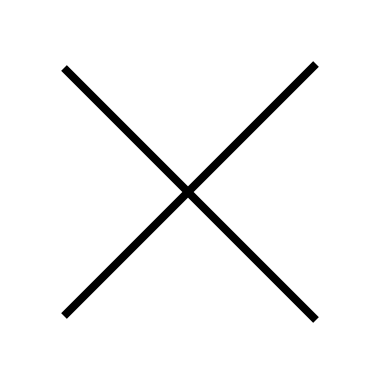 | 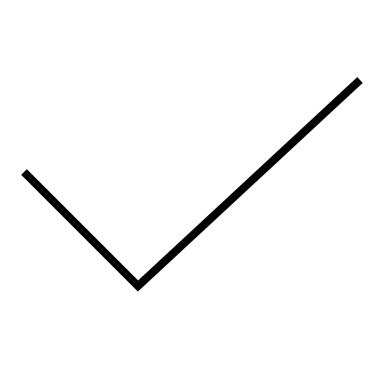 | NS | NA | 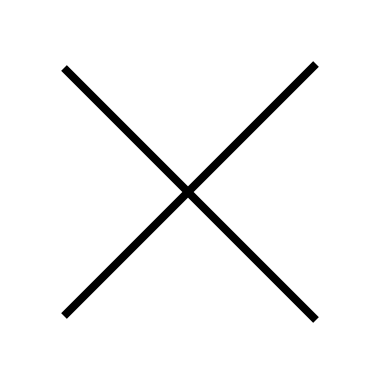 | F |
| Callaghan | 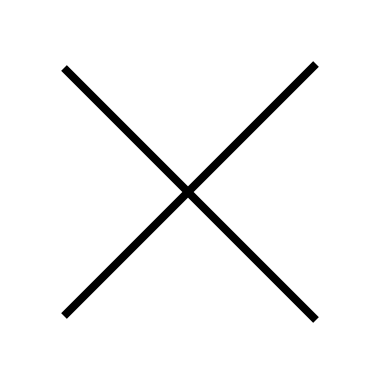 | 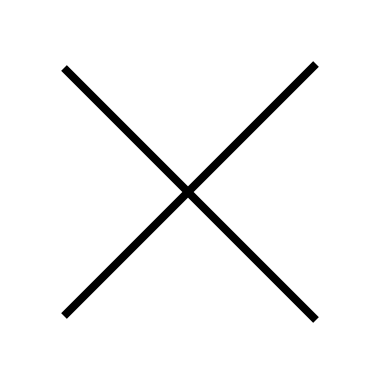 | 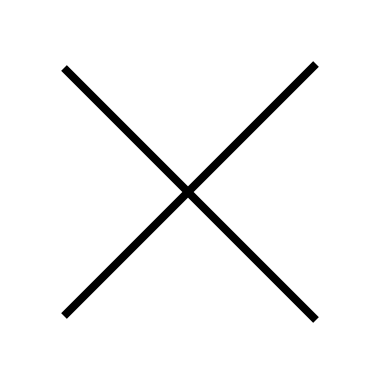 | 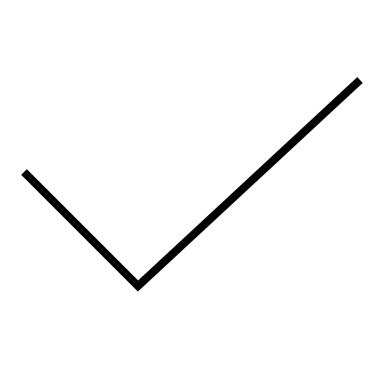 | 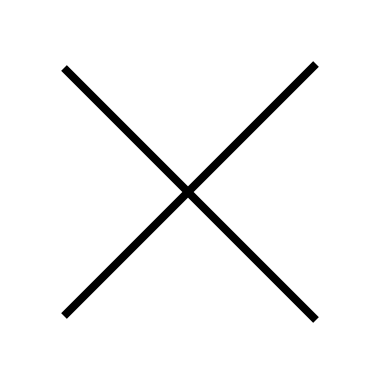 | 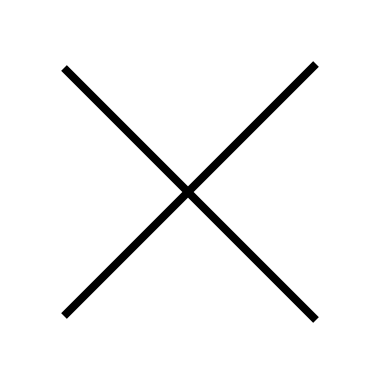 | 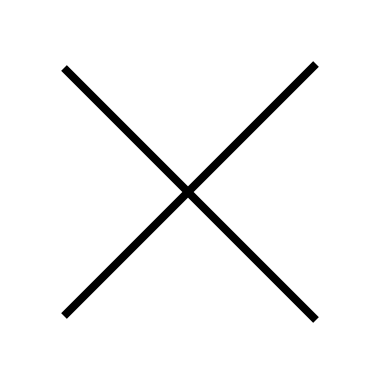 | 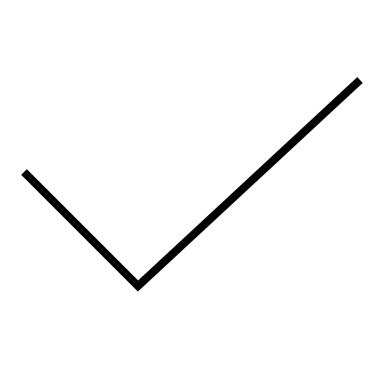 | 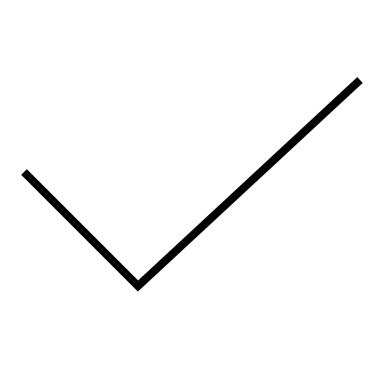 | 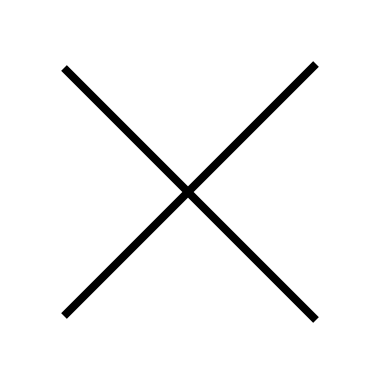 | 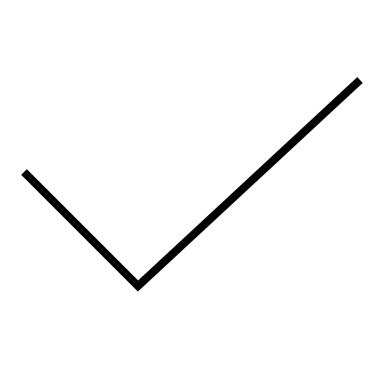 | NS | NA | 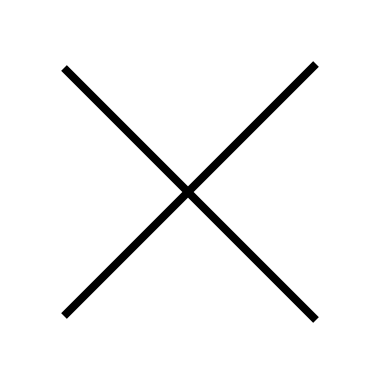 | P |
| Carlson 2018 | 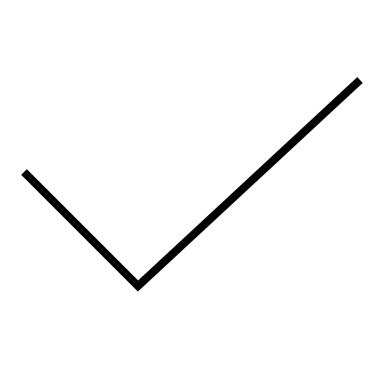 | 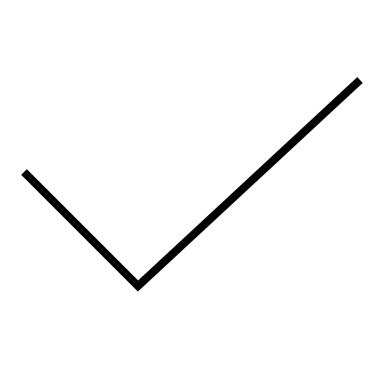 | 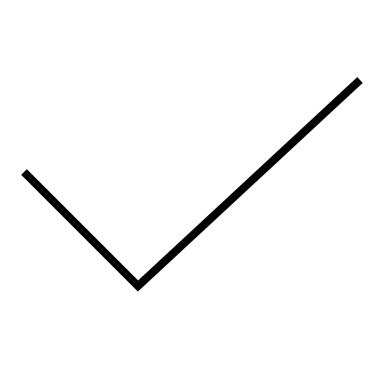 | 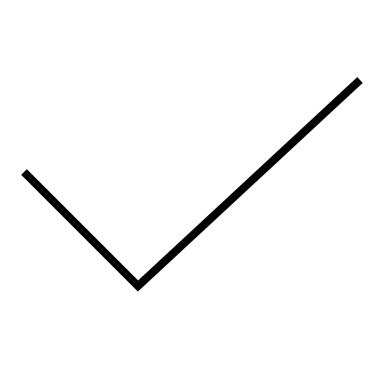 | 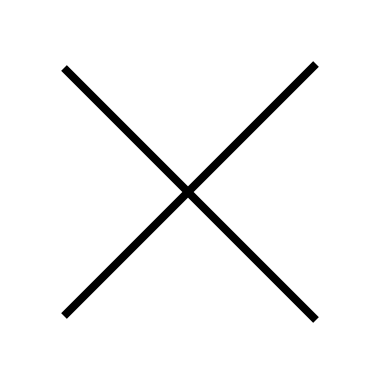 | 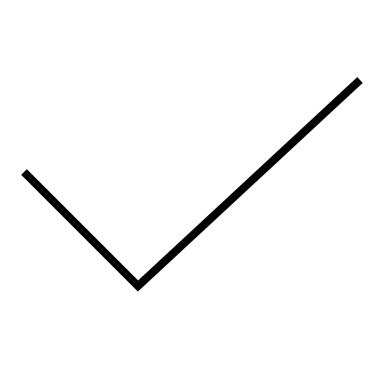 | 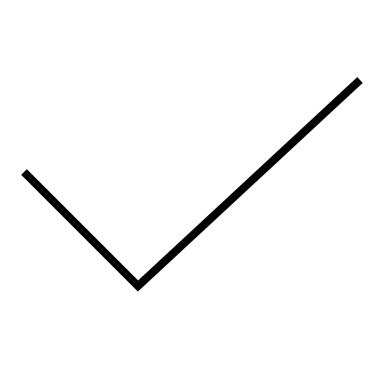 | 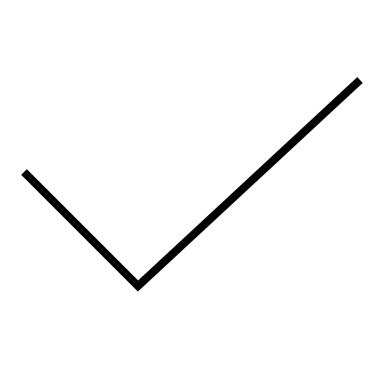 | 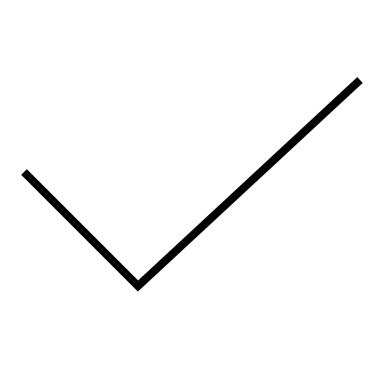 | 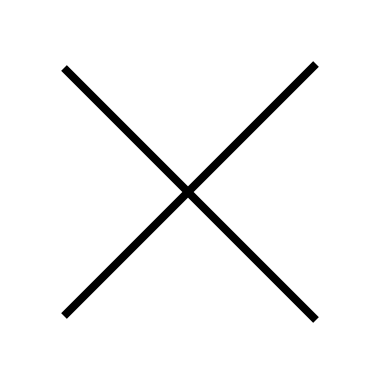 | 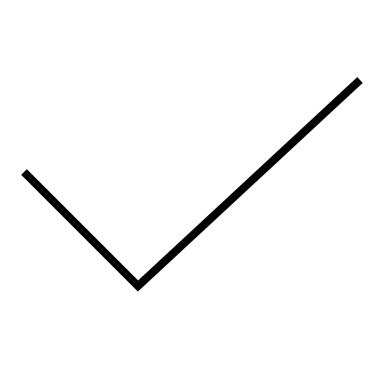 | 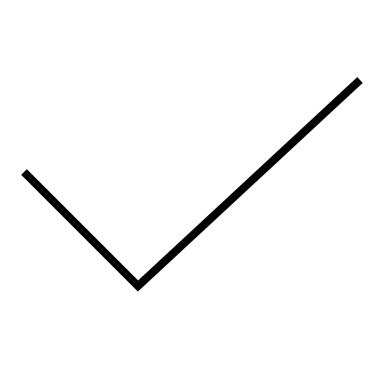 | 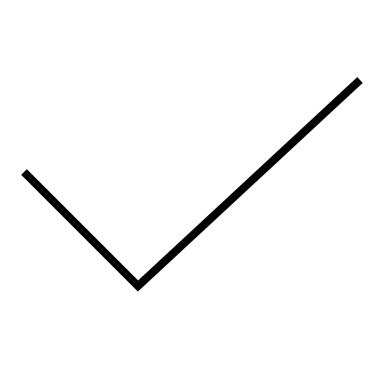 | 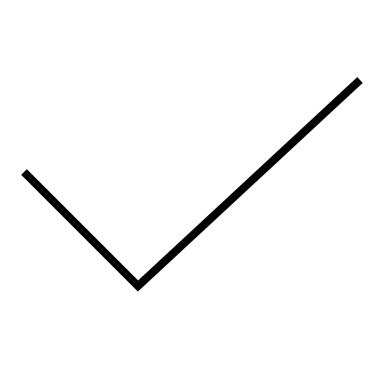 | G |
| Carlson 2021 | 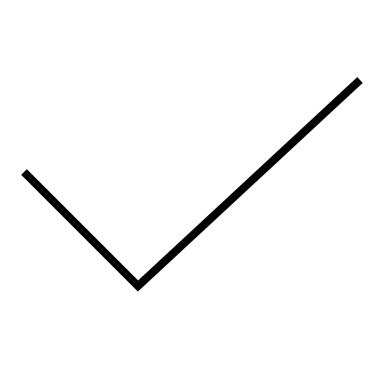 | 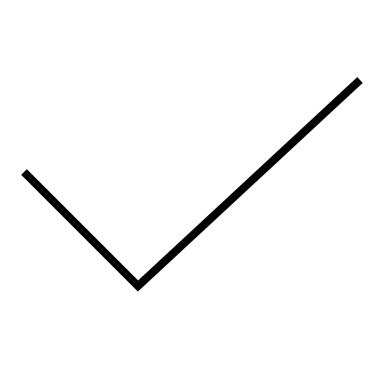 | NS | 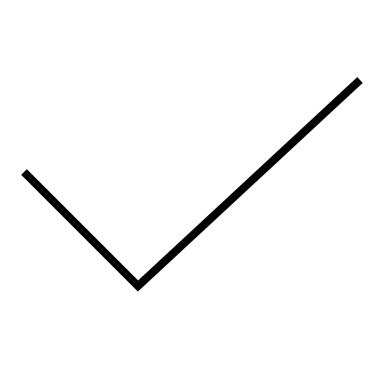 | 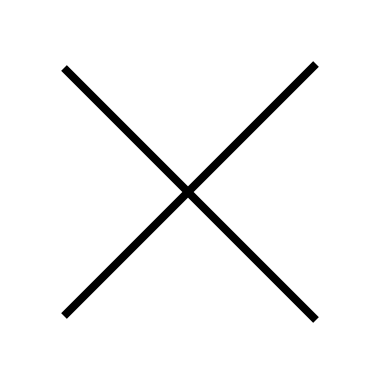 | 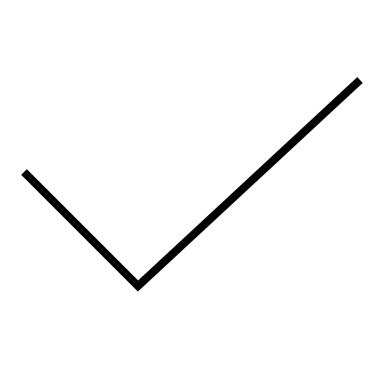 | 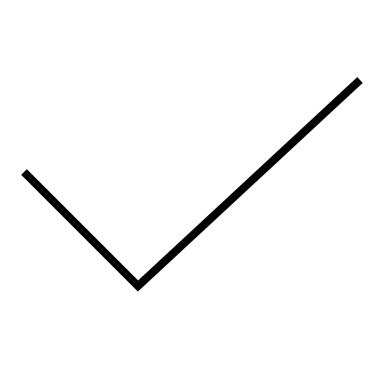 | 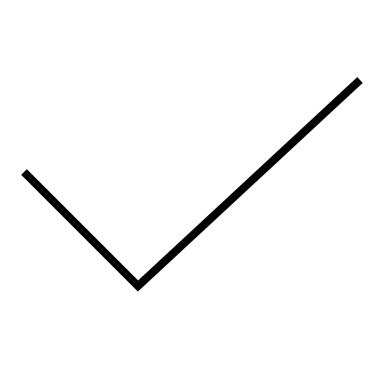 | 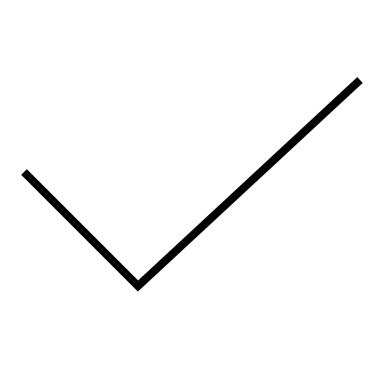 | 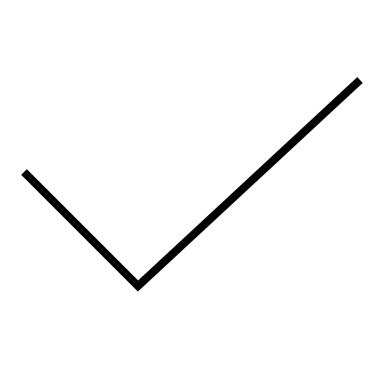 | 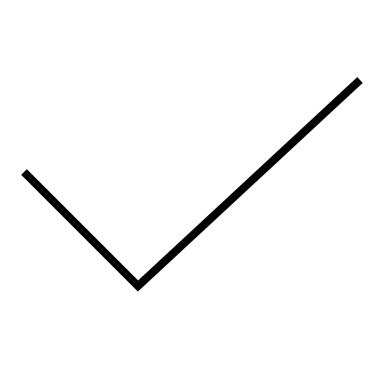 | 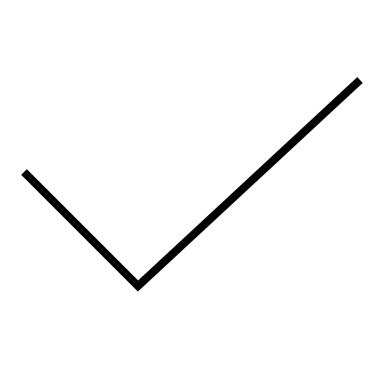 | 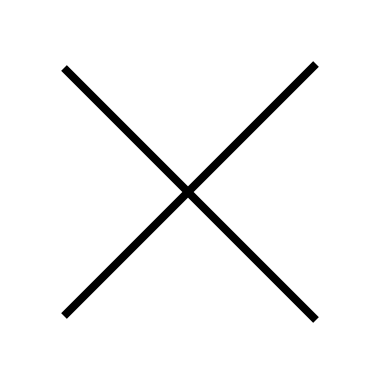 | 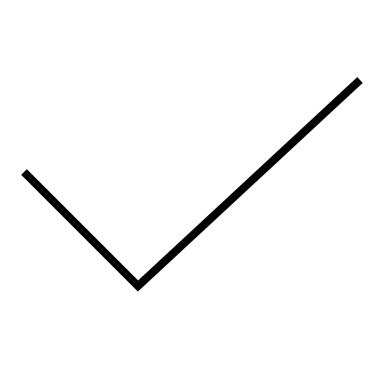 | G |
| Christian | 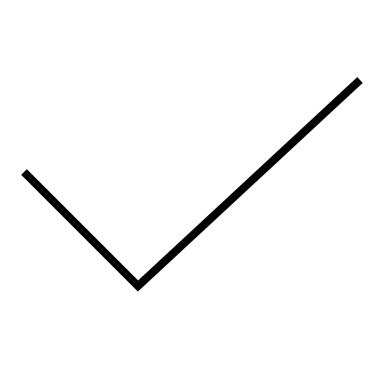 | 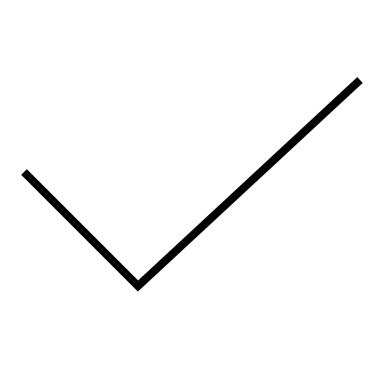 | NA | 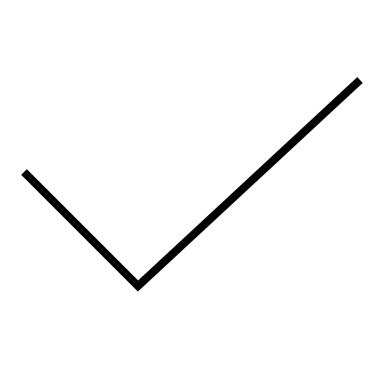 | 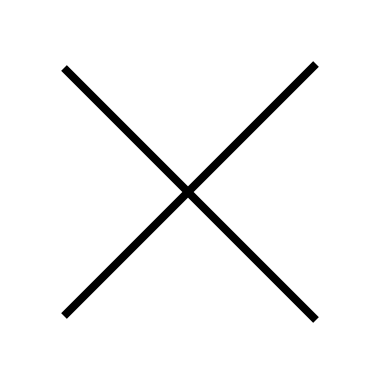 | 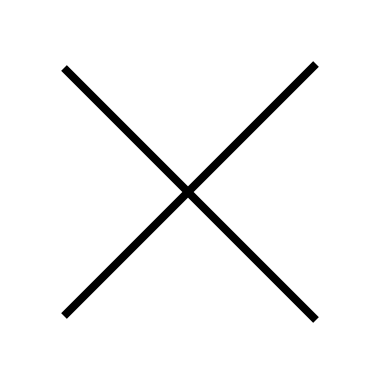 | 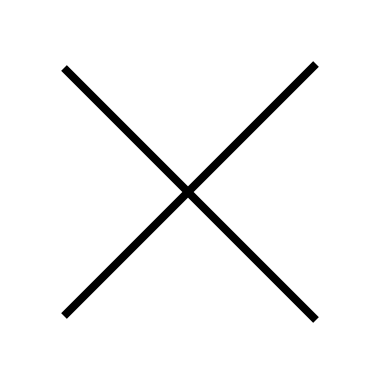 | 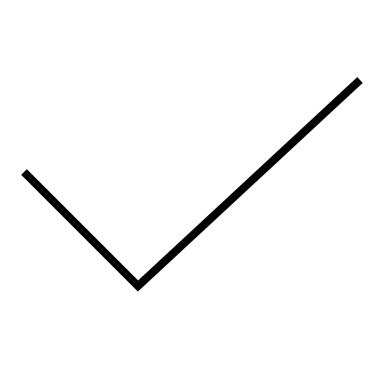 | 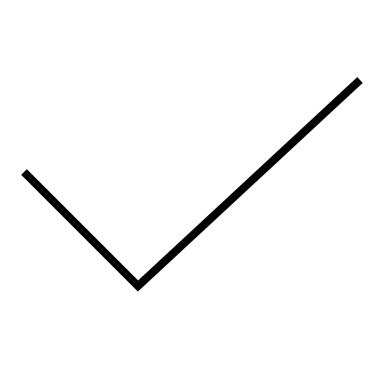 | 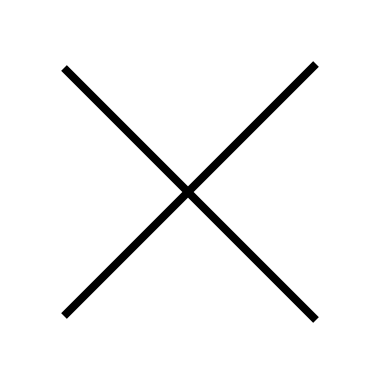 | 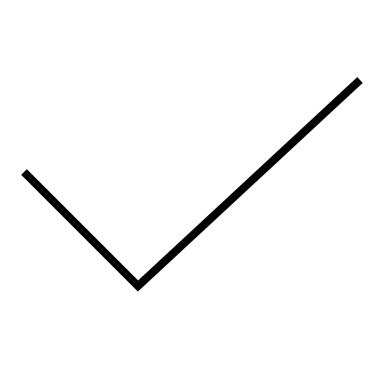 | NS | NA | 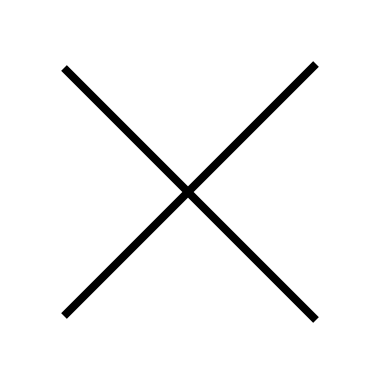 | F |
| Flannery | 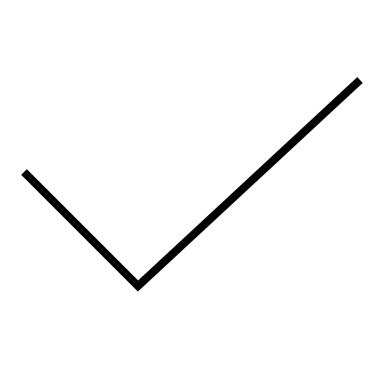 | 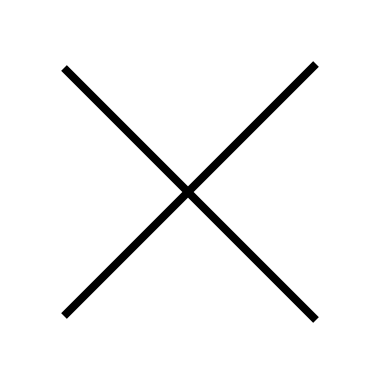 | 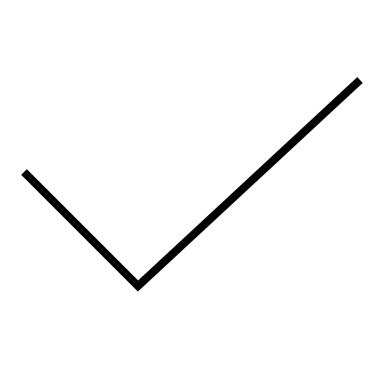 | 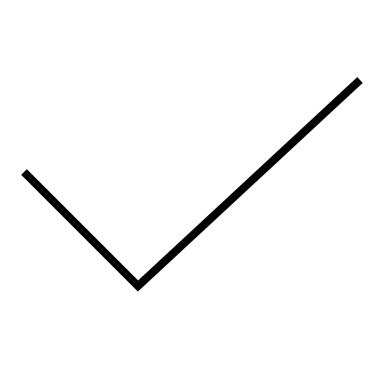 | 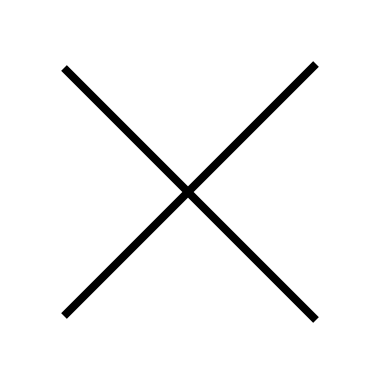 | 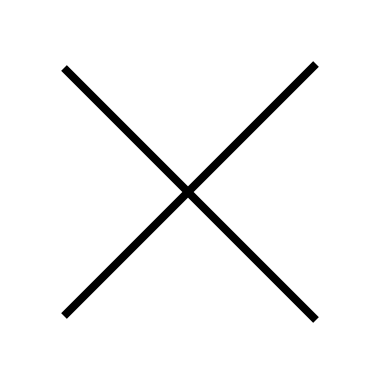 | 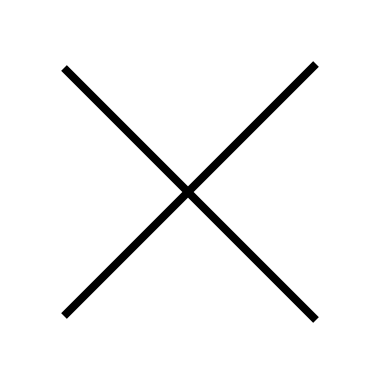 | 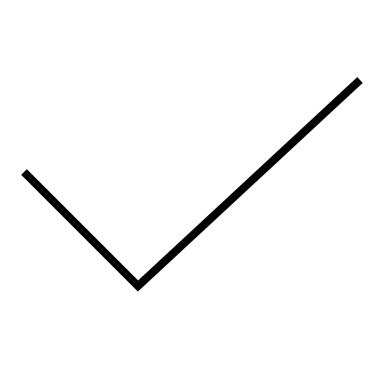 | 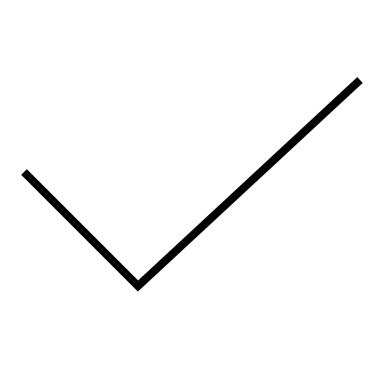 | 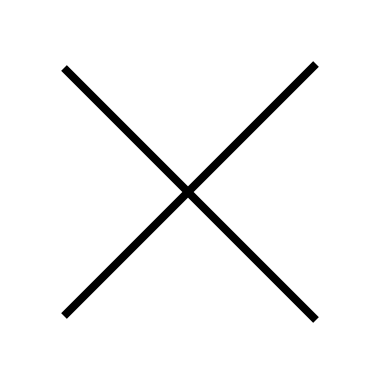 | 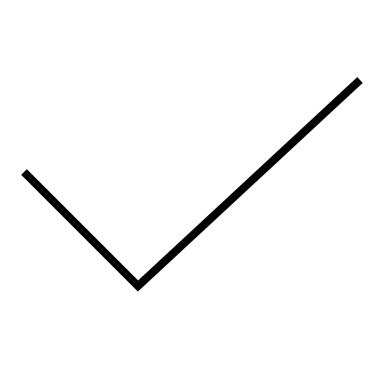 | NS | NA | 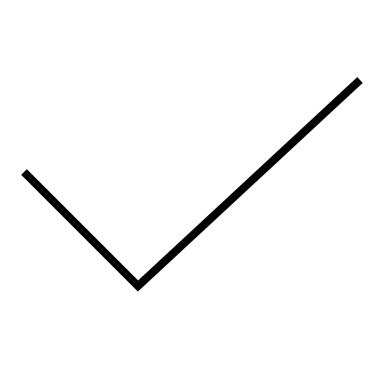 | F |
| Fox | 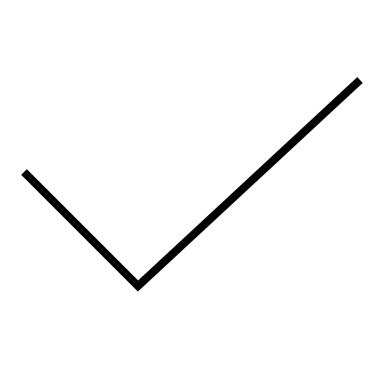 | 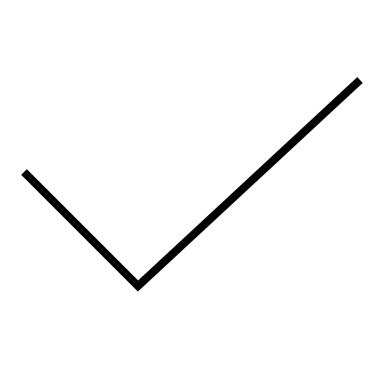 | 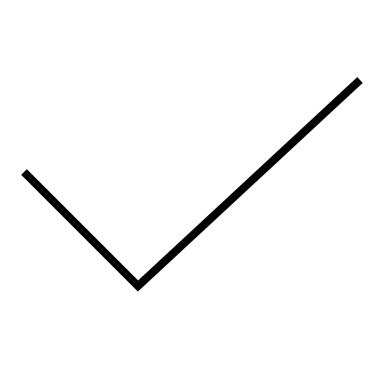 | 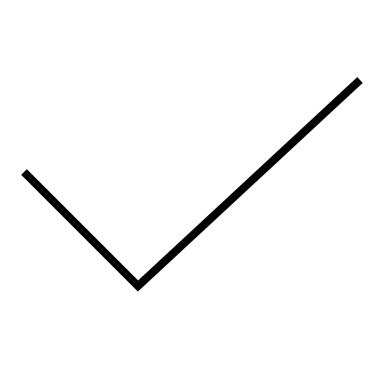 |  |  |  |  |  |  |  |  | NS |  | G |
| Gao |  |  |  |  |  |  |  |  |  |  |  |  |  |  | F |
| Guzzardi |  |  |  |  |  |  |  |  |  |  |  | NS |  |  | F |
| Kelsey |  |  | NA |  |  |  |  |  |  |  |  | NS | NA |  | F |
| Loughman |  |  |  |  |  |  |  |  |  |  |  | NS | NS |  | G |
| Michels 2019 |  |  |  |  |  |  |  |  |  |  |  | NS | NA |  | F |
| Rothenberg |  |  |  |  |  |  |  |  |  |  |  | NS | NA |  | F |
| Sordillo |  |  |  |  |  |  |  |  |  |  |  | NS |  |  | F |
| Streit |  |  |  |  |  |  |  |  |  |  |  | NS | NA |  | F |
| Tamana |  |  |  |  |  |  |  |  |  |  |  | NS |  |  | G |
| Wang |  |  |  |  |  |  |  |  |  |  |  | NS | NA |  | F |
| Zhang |  |  |  |  |  |  |  |  |  |  |  | NS | NA |  | F |

Quality was rated as P for poor (0–4 out of 14 questions), F for fair (5–10 out of 14 questions), or G for good (11–14 out of 14 questions); NA: not applicable, NS: not specified

**Supplemental Table 5.** Quality of the included studies by the NIH Quality Assessment Tool for controlled intervention studies.

| **Study** | 1. Was the study described as randomized, a randomized trial, a randomized clinical trial, or an RCT? |  | 2. Was the method of randomization adequate (i.e., use of randomly generated assignment)? | 3. Was the treatment allocation concealed (so that assignments could not be predicted)? | 4. Were study participants and providers blinded to treatment group assignment? | 5. Were the people assessing the outcomes blinded to the participants' group assignments? | 6. Were the groups similar at baseline on important characteristics that could affect outcomes (e.g., demographics, risk factors, co-morbid conditions)? | 7. Was the overall drop-out rate from the study at endpoint 20% or lower of the number allocated to treatment? | 8. Was the differential drop-out rate (between treatment groups) at endpoint 15 percentage points or lower? | 9. Was there high adherence to the intervention protocols for each treatment group? | 10. Were other interventions avoided or similar in the groups (e.g., similar background treatments)? | 11. Were outcomes assessed using valid and reliable measures, implemented consistently across all study participants? | 12. Did the authors report that the sample size was sufficiently large to be able to detect a difference in the main outcome between groups with at least 80% power? | 13. Were outcomes reported or subgroups analyzed prespecified (i.e., identified before analyses were conducted)? | 14. Were all randomized participants analyzed in the group to which they were originally assigned, i.e., did they use an intention-to-treat analysis? | Summary Quality |
| --- | --- | --- | --- | --- | --- | --- | --- | --- | --- | --- | --- | --- | --- | --- | --- | --- |
| Sobko |  |  |  |  |  |  |  |  |  |  |  |  |  |  |  | G |
| Wu |  |  |  |  | NS | NS |  |  |  |  |  |  |  |  |  | P |

Quality was rated as P for poor, F for fair, or G for good; NA: not applicable, NS: not specified.
